# Profound DNA methylomic differences between single- and multi-fraction alpha irradiations of lung fibroblasts

**DOI:** 10.1186/s13148-023-01564-z

**Published:** 2023-10-27

**Authors:** Marilyn N. Vera-Chang, John M. Danforth, Marilyne Stuart, Aaron A. Goodarzi, Marjorie Brand, Richard B. Richardson

**Affiliations:** 1 Radiobiology and Health Branch, Chalk River Laboratories, Canadian Nuclear Laboratories, Chalk River, ON K0J 1J0 Canada; 2 Departments of Biochemistry and Molecular Biology and Oncology, Cumming School of Medicine, Robson DNA Science Centre, Charbonneau Cancer Institute, University of Calgary, Calgary, AB T2N 1N4 Canada; 3 Environment and Waste Technologies Branch, Chalk River Laboratories, Canadian Nuclear Laboratories, Chalk River, ON K0J 1J0 Canada; 4 Ottawa Hospital Research Institute, Ottawa, ON K1H 8L6 Canada; 5 Department of Cellular and Molecular Medicine, University of Ottawa, Ottawa, ON K1H 8L6 Canada; 6 McGill Medical Physics Unit, Cedars Cancer Centre-Glen Site, Montreal, QC H4A 3J1 Canada

**Keywords:** Alpha particles, Radon, Lung, Methylome, Aging, Cancer, Mitochondria, Radiation

## Abstract

**Background:**

Alpha (α)-radiation is a ubiquitous environmental agent with epigenotoxic effects. Human exposure to α-radiation at potentially harmful levels can occur repetitively over the long term via inhalation of naturally occurring radon gas that accumulates in enclosed spaces, or as a result of a single exposure from a nuclear accident. Alterations in epigenetic DNA methylation (DNAm) have been implicated in normal aging and cancer pathogenesis. Nevertheless, the effects of aberrations in the methylome of human lung cells following exposure to single or multiple α-irradiation events on these processes remain unexplored.

**Results:**

We performed genome-wide DNAm profiling of human embryonic lung fibroblasts from control and irradiated cells using americium-241 α-sources. Cells were α-irradiated in quadruplicates to seven doses using two exposure regimens, a single-fraction (SF) where the total dose was given at once, and a multi-fraction (MF) method, where the total dose was equally distributed over 14 consecutive days. Our results revealed that SF irradiations were prone to a decrease in DNAm levels, while MF irradiations mostly increased DNAm. The analysis also showed that the gene body (i.e., exons and introns) was the region most altered by both the SF hypomethylation and the MF hypermethylation. Additionally, the MF irradiations induced the highest number of differentially methylated regions in genes associated with DNAm biomarkers of aging, carcinogenesis, and cardiovascular disease. The DNAm profile of the oncogenes and tumor suppressor genes suggests that the fibroblasts manifested a defensive response to the MF α-irradiation. Key DNAm events of ionizing radiation exposure, including changes in methylation levels in mitochondria dysfunction-related genes, were mainly identified in the MF groups. However, these alterations were under-represented, indicating that the mitochondria undergo adaptive mechanisms, aside from DNAm, in response to radiation-induced oxidative stress.

**Conclusions:**

We identified a contrasting methylomic profile in the lung fibroblasts α-irradiated to SF compared with MF exposures. These findings demonstrate that the methylome response of the lung cells to α-radiation is highly dependent on both the total dose and the exposure regimen. They also provide novel insights into potential biomarkers of α-radiation, which may contribute to the development of innovative approaches to detect, prevent, and treat α-particle-related diseases.

**Graphical abstract:**

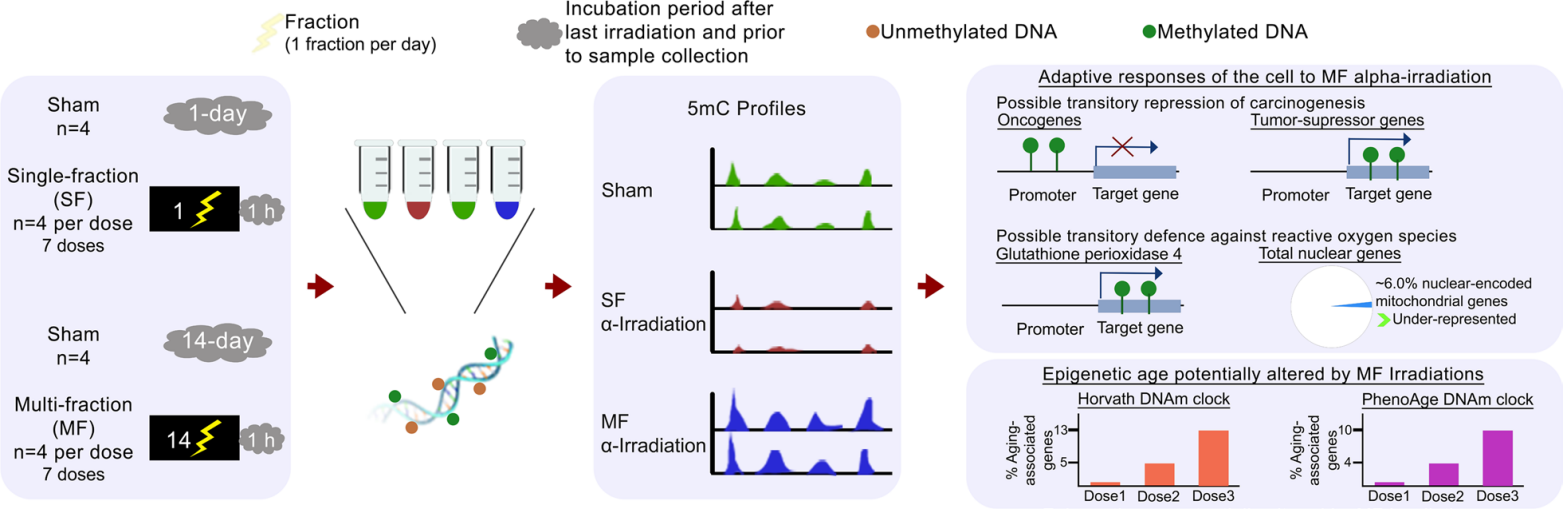

**Supplementary Information:**

The online version contains supplementary material available at 10.1186/s13148-023-01564-z.

## Background

Alpha (α)-particle radiation is a ubiquitous naturally occurring environmental carcinogen. Modern construction practices [1–3], activity patterns, and shifting behaviors [4–7] are among the main factors contributing to the increase in indoor human exposure to α-radiation, rendering it a major contemporary health concern. The α-emitter radon-222 (^222^Rn), a radioactive noble gas derived from the decay chain of uranium and radium, naturally present in rocks and soil, can accumulate to harmful levels in enclosed spaces within the residential and occupational built environments. The inhalation of ^222^Rn and its progeny constitutes the major source of human exposure to ionizing radiation [1]. A 2021 study by Simms et al. [7] estimated that the geometric mean annual α-particle radiation dose rate to lungs resulting from Canadian residential ^222^Rn exposure was 4.08 mSv·y^−1^ (min 0.08 mSv·y^−1^; max 169 mSv·y^−1^), corresponding to a mean cumulative dose of 290 mSv (min 5.7 mSv; max 12,000 mSv) over an average lifespan of 71 years [8]. The authors further reported that ^222^Rn dose rates exposure from residences built in the twenty-first century (mean 5.01 mSv·y^−1^) were higher than those built in the twentieth century (mean 3.45–4.22 mSv·y^−1^). In a separate study [5], radiation doses from ^222^Rn were also found to be highly dependent on human behavior and decision-making, with people who delayed or declined reducing high residential ^222^Rn levels experiencing a mean dose rate of 10.3 mSv.y^−1^, while those who quickly took ^222^Rn reduction action decreased their exposure to 0.75 mSv.y^−1^. Notably, a study from 2023 found a 19% increase in annual radiation doses from Canadian residential ^222^Rn exposure as a result of the COVID-19 pandemic [4]. This higher dose was dependent on factors such as age, community type, and occupational status. Exposure to α-particles can occur not only in residential environments but also through medical treatments [9, 10], occupational settings [11, 12], and nuclear incidents [13].

Exposure to ^222^Rn has been extensively associated with an increased risk of carcinogenesis [14–16]. Epidemiological studies have shown a higher incidence of lung cancer among underground uranium miners who are known to be occupationally exposed to ^222^Rn [17]. These findings were further confirmed by the 1998 Biological Effects of Ionizing Radiation (BEIR) VI Report, which identified ^222^Rn as the second leading cause of lung cancer, following tobacco smoking. Inhalation is the primary route through which ^222^Rn enters the body, and upon decay, it emits four α-particles before reaching its stable end product lead-206 [18]. These particles deposit their ionization energy within lung cells, potentially causing the displacement of electrons and rendering molecules charged. DNA is highly susceptible to such ionization events, and hence, aberrant gene expression resulting from direct DNA damage is the most understood molecular mechanism by which α-radiation exerts its biological effects [19–22]. Epigenetic processes, which play a key role in modulating gene expression during cellular development and differentiation, are known to regulate molecular activity in response to various stressors, including environmental insults such as ionizing radiation [23]. These epigenetic events may potentially be transmitted, via the germline, to subsequent generations [24].

Epigenetic modifications, predominantly DNA methylation (DNAm), have become a primary focus in cancer research for their role in cancer initiation and development [25, 26]. DNA methylation is the DNA methyltransferase-catalyzed covalent addition of a methyl group to the fifth carbon of the cytosine residue within cytosine and guanine (CpG) dinucleotides, forming 5-methylcytosine (5mC). Mammalian somatic cells contain approximately 28 million CpG sites [27], of which 70 to 80% are methylated [28]. Gain in 5mC levels at gene promoters usually results in decreased gene expression, whereas gene body methylation tends to induce the opposite effect [29]. Contrary to healthy cells, cancer cells display a global reduction in DNAm levels, except at the promoter sites of tumor suppressor genes, where an increase in 5mC levels is exhibited [30]. Thus far, the few studies reporting on DNAm abnormalities following α-exposure used assays specific to cancer-associated genes [11, 31]. However, no information on the effects of low (≤ 0.5 Gy) or high (> 0.5 Gy) doses of α-radiation on the whole methylome of lung cells was available until this current study.

In addition to carcinogenesis, it has been strongly suggested that epigenetic variations may underlie the cellular deterioration of aging [32]. This breakthrough led to the identification of molecular markers of aging based on DNAm data that can accurately estimate the biological age of any tissue in the human body [33, 34]. These DNAm-based age estimators are known as epigenetic aging clocks. *Chronological age* represents the actual time an individual has been alive, whereas *biological age* is specific to each individual and is estimated based on physiological parameters and cellular health status. Biological age is, therefore, defined as the age reflecting disease processes and mortality [35]. The methylome lends itself to differentiation between healthy and unhealthy aging leading to a decline in cognitive, metabolic, and physiological abilities [36]. Since environmental agents have the potential to alter the epigenome and DNAm can modulate health outcomes, it has been proposed that certain environmental stressors can accelerate the biological age of an individual which is reflected in the DNAm biological clock [37].

Interestingly, the hypothesis that ionizing radiation could accelerate aging originated in the late 1940s upon observing a dose-dependent life-shortening effect in irradiated rodents [38]. However, this assumption was disregarded in the 1970s as it was noted that among the 82,000 Japanese A-bomb survivors being followed for mortality, 17.6% of deaths were attributed to non-neoplastic diseases. This event generated controversies as to whether these diseases were induced by radiation or by a radiation-independent mechanism [39]. Since then, there have been numerous studies linking ionizing radiation to accelerated aging [40], but analysis with a focus on the epigenome has been rarely conducted.

In order to simulate the environmental exposure to ^222^Rn, this study used normal human lung fibroblast cells derived from a 3-month-old female, as the lungs are the primary target following inhalation of this hazard. Here, we performed a comprehensive comparative DNA methylome analysis between non-irradiated and irradiated fibroblasts, with a particular focus on both cancer-related and aging-associated effects. To account for the variability in worldwide human exposure to α-radiation, including both low and high doses and single and multiple exposures, we used a range of seven doses of α-radiation. Each dose was administered using two exposure regimens: a single-fraction (SF) approach, where the total dose was delivered in a single session, and a multi-fraction (MF) method, where the same dose was delivered over fourteen consecutive days in equal daily fractions. The SF irradiation regimen enabled us to better understand the effects of α-exposure resulting from a potential dirty bomb detonation or a nuclear incident, whereas the MF groups represented individuals exposed in residential, radiotherapeutic, and occupational settings. Our findings contribute to advancing the understanding of how both dosage levels and exposure regimens affect the methylome response to α-radiation. Furthermore, they provide novel insights into potential biomarkers of α-radiation, which may contribute to the development of innovative approaches to prevent, detect, and treat α-related diseases.

## Results

### Contrasting effects of SF and MF α-irradiations on the DNA methylome landscape

To investigate the DNA methylomic effects instigated by α-particles in lung cells, we performed in vitro α-irradiations on human WI-38 cells, a normal fibroblast cell line derived from a 3-month-old female lung tissue. To ensure a comprehensive analysis, cells were exposed to a wide range of human-relevant doses of α-irradiations. Given that previous studies have demonstrated differences between SF and MF regimens' effects on health outcomes, particularly in optimizing radiation treatment for patients with cancer [41, 42], we administered each dose using both SF and MF exposures. DNA methylation levels were subsequently assessed using methylated DNA immunoprecipitation coupled with deep sequencing (MeDIP-seq), allowing us to identify a great number of differentially methylated regions (DMRs) between the controls and each of the irradiated groups (Additional file 1: Table S3). For simplicity, the fibroblasts irradiated with SF treatment doses of 2.0, 11, 23, 110, 220, 1,100, and 2,200 mGy at a dose rate as described (Additional file 1: Table S1) will be referred to as SF^2.0^, SF^11^, SF^23^, SF^110^, SF^220^, SF^1100^, and SF^2200^, respectively. Similarly, cells exposed to MF doses of 0.14, 0.79, 1.6, 7.9, 16, 79, and 160 mGy·day^−1^ for 14 days every 24 h at a dose rate as described (Additional file 1: Table S2) will be denoted by the total delivered dose: MF^2.0^, MF^11^, MF^23^, MF^110^, MF^220^, MF^1100^, and MF^2200^, respectively.

**Fig. 1:**
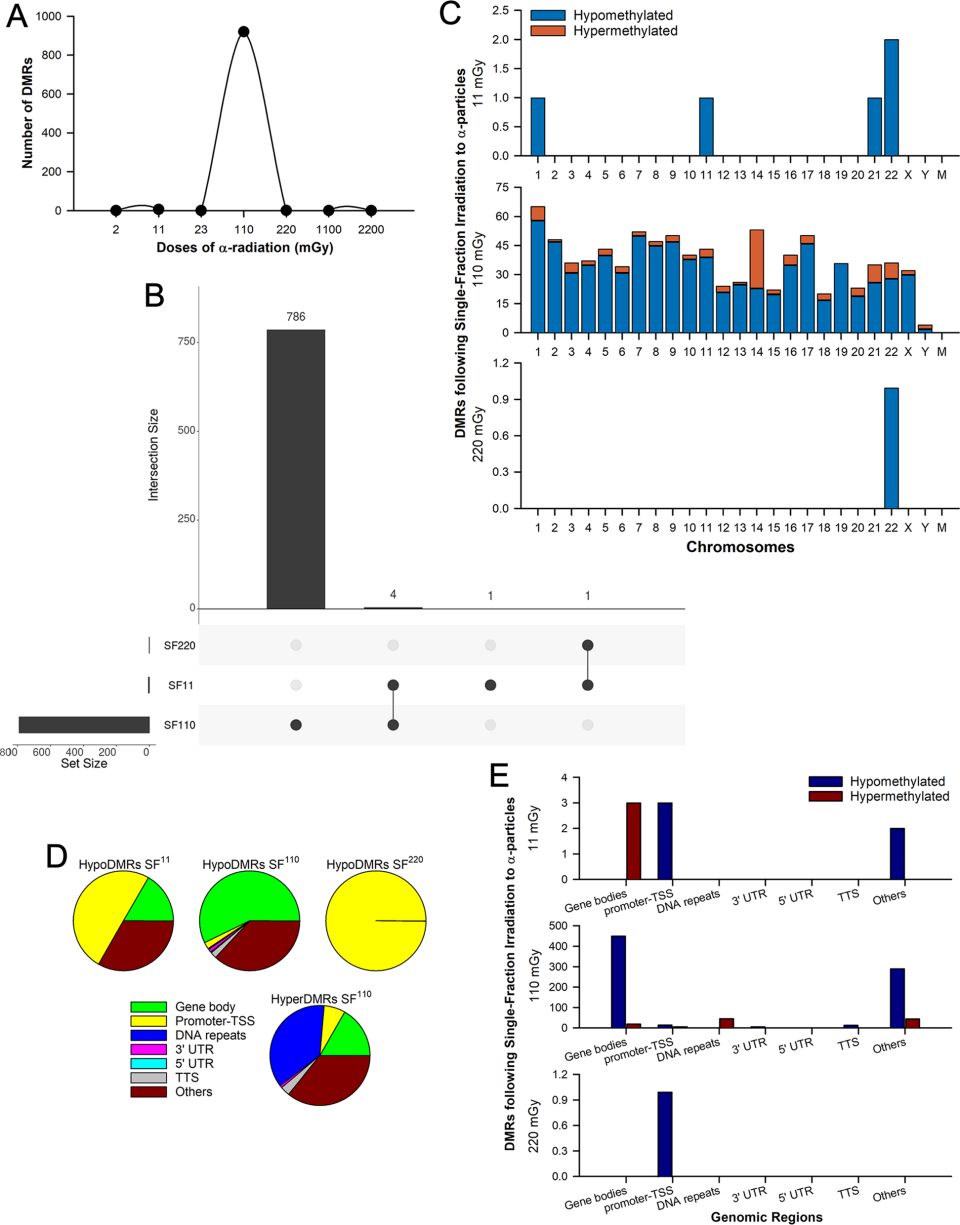
Differentially methylated regions (DMRs) in lung fibroblasts following irradiation to single-fraction (SF) doses of α-particles. **A** Total number of DMRs identified using the Bioconductor MEDIPS package. **B** UpSet plot showing the number of the identified DMRs in the fibroblasts across the different SF α-irradiation doses. **C** Bar chart displaying the number of hypo and hyperDMRs across all chromosomes. **D** Percentages of the genomic regions that were found to be differentially enriched in each irradiation group. **E** Bar chart displaying the number of hypo and hyperDMRs identified in each genomic region. Each irradiation group includes four biological replicates per dose, each independently irradiated and assessed. Abbreviations: hypoDMRs, hypomethylated DMRs; hyperDMRs, hypermethylated DMRs; TSS, transcription start site; TTS, transcription termination site; 3’ UTR, 3’ untranslated region; and 5’ UTR, 5’ untranslated region

Methylome analysis revealed a non-monotonic dose–response pattern in the fibroblasts exposed to SF irradiations. Among the SF doses that were tested in this study, only the medium dose of SF^110^ resulted in a large number of DNAm changes, with 920 DMRs detected in the α-irradiated cells compared with the control (Fig. 1a, b). In contrast, the low and high doses SF^2.0^, SF^11^, SF^23^, SF^220^, SF^1100^, and SF^2200^ induced minimal effects, with only 0, 6, 0, 1, 0, and 0 DMRs, respectively (Additional file 1: Table S3). The most prevalent alteration observed in the SF groups was hypomethylation, accounting for 86.9% of the total changes. Hypomethylated DMRs (hypoDMRs) were observed in 100% of the SF^11^ (6/6) and SF^220^ (1/1) groups, and in 85.8% (789/920) of the SF^110^ group (Fig. 1c). Further analysis of the DNAm levels on individual chromosomes is illustrated in Fig. 1c. Furthermore, our analysis identified the body region of the gene as the location of the genome harboring the highest percentage (57%) of hypoDMRs (Fig. 1d, e).

**Fig. 2:**
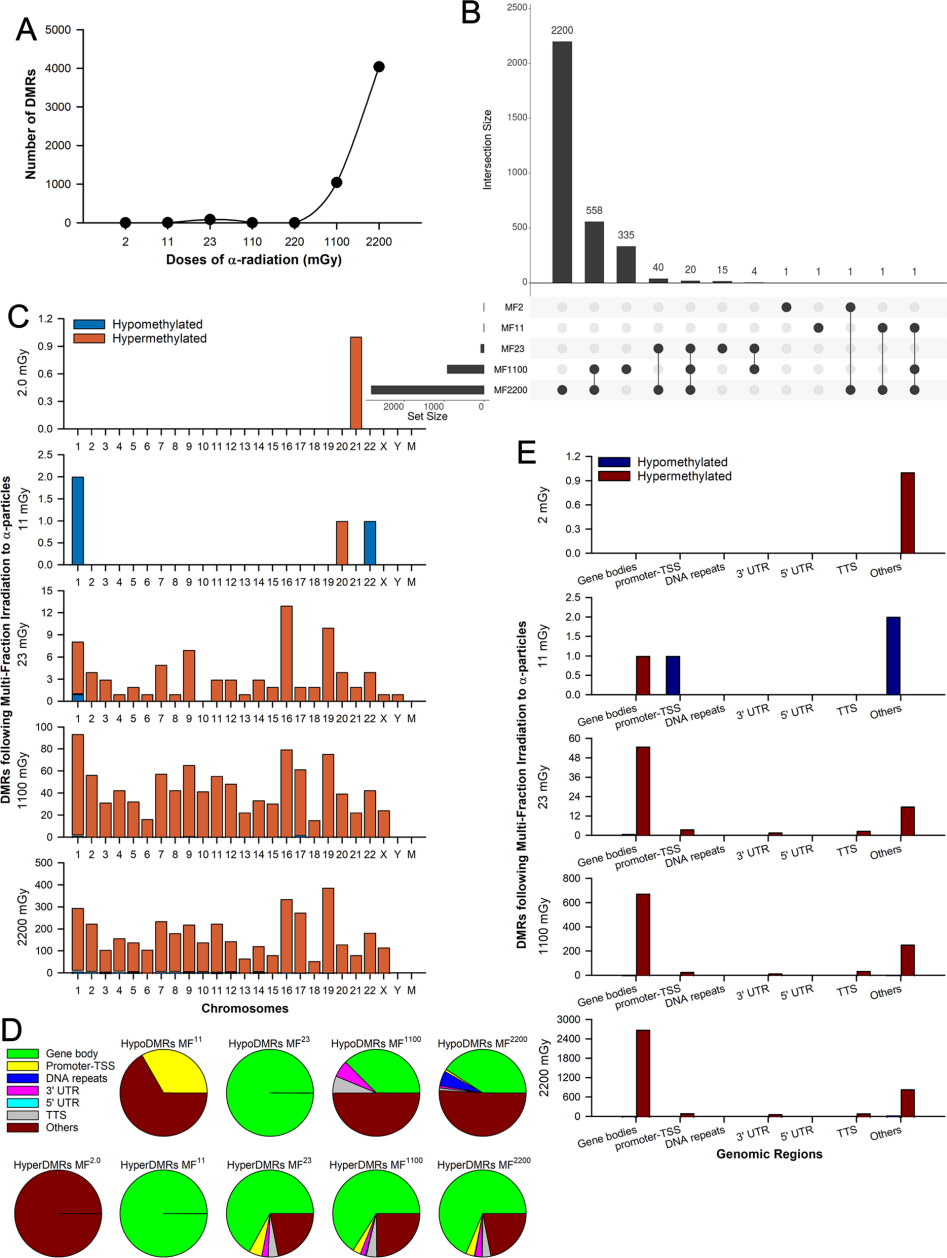
Differentially methylated regions (DMRs) in lung fibroblasts following irradiation to multi-fraction (MF) doses of α-particles. **A** Total number of DMRs identified using the Bioconductor MEDIPS package. **B** UpSet plot showing the number of the identified DMRs in the fibroblasts across the different MF α-irradiation doses. **C** Bar chart displaying the number of hypo and hyperDMRs across all chromosomes. **D** Percentages of the genomic regions that were found to be differentially enriched in each irradiation group. **E** Bar chart displaying the number of hypo and hyperDMRs identified in each genomic region. Each dose of ionizing radiation was equally distributed every 24 h over 14 consecutive days. Each irradiation group includes four biological replicates per dose, each independently irradiated and assessed. Abbreviations: hypoDMRs, hypomethylated DMRs; hyperDMRs, hypermethylated DMRs; TSS, transcription start site; TTS, transcription termination site; 3’ UTR, 3’ untranslated region; and 5’ UTR, 5’ untranslated region

In contrast to the effects of SF exposures on the methylome, the MF α-irradiations induced a dose–response relationship following a quadratic polynomial function (R^2^ = 0.999) in which the number of DMRs increased with the dose (Fig. 2a, b). Specifically, the doses MF^2.0^, MF^11^, MF^23^, MF^1100^, and MF^2200^ led to 1, 4, 83, 1,044, and 4,039 DMRs, respectively (Additional file 1: Table S3). The majority of DMRs resulting from the MF α-irradiations were hypermethylated with the following distribution, 100% (1/1) in the MF^2.0^ group, 25% (1/4) in the MF^11^ group, 98.8% (82/83) in the MF^23^ group, 98.5% (1,028/1,044) in the MF^1100^ group, and 97.3% (3,929/4,039) in the MF^2200^ group (Fig. 2c). Distribution of the identified DMRs across all chromosomes is represented in Fig. 2c. The gene body region was the primary target of the hypermethylated DMRs (hyperDMRs) induced by the MF treatments, accounting for 68% of these alterations (Fig. 2d, e).

## Aging-associated DNAm events were mainly identified in the MF α-irradiated fibroblasts

The next steps were to examine the presence of aging-associated epigenetic marks in the SF and MF α-irradiated fibroblasts. Established algorithms based on DNAm (DNAm clocks) were predominantly profiled and developed using human DNAm arrays [33, 34, 43–46], which identify 5mC alterations at predefined CpG sites across the genome. These CpG sites from the DNAm clocks were specifically selected from genes that were found to consistently undergo DNAm changes with aging. As an alternative approach to identifying epigenetic biomarkers of aging, we compared our DMRs-associated genes obtained from the MeDIP-seq experiment, with the gene lists derived from two well-established epigenetic DNAm clock algorithms, namely Horvath’s epigenetic aging clock [33] and Levine and colleagues’ biological clock known as PhenoAge [34]. These clocks have shown robust correlations with age independently of tissue type. Horvath’s clock is based on the DNAm levels of 353 CpG sites, potentially controlling the expression of 344 nearby aging-related genes [33]. Although primarily developed to predict an individual’s DNAm age, Horvath’s epigenetic aging clock can also be used as a predictor of age-related diseases. Conversely, PhenoAge is a better estimator of mortality risk and relies on measurements from 513 CpG sites, potentially controlling the expression of 505 nearby aging-related genes.

**Fig. 3:**
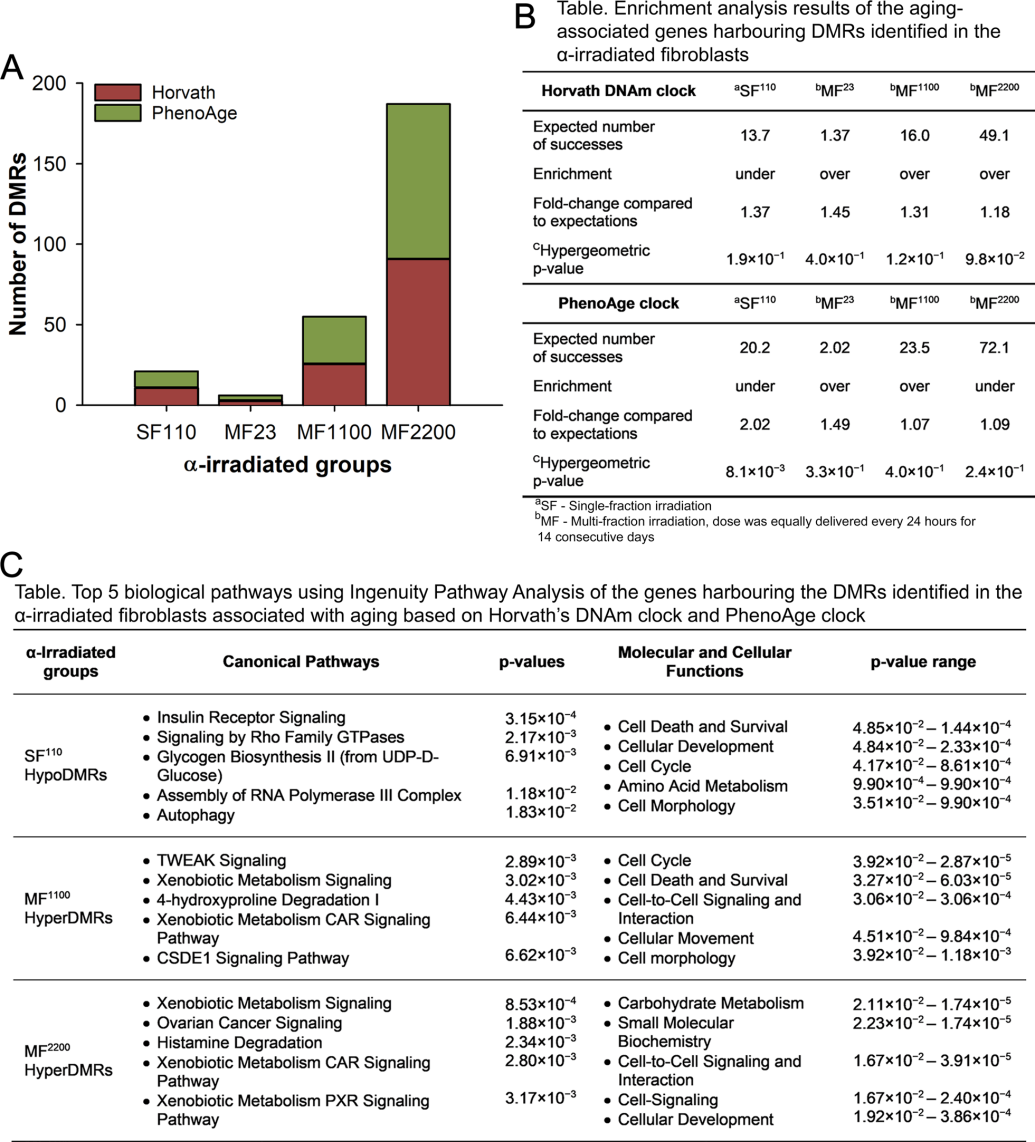
Aging-associated DNA methylation (DNAm) events identified in the α-irradiated lung fibroblasts. These events are based on the DNAm clock from Horvath [33] and on the PhenoAge DNAm clock from Levine et al. [34]. **A** Number of differentially methylated regions (DMRs) from the α-irradiated fibroblasts whose neighboring genes were associated with aging as established by the two selected DNAm clocks. **B** Table displaying the enrichment analysis results of the aging-associated genes whose DNAm levels were altered in the α-irradiated fibroblasts, computed using the cumulative distribution function of the hypergeometric equation from [49]. **C** Top 5 functional classification of the genes affected by the aging-associated DMRs using Ingenuity Pathway Analysis. Abbreviations: SF, single-fraction; MF, multi-fraction; TSS, transcription start site; TTS, transcription termination site; 3’ UTR, 3’ untranslated region; and 5’ UTR, 5’ untranslated region

Analysis of the SF-irradiated groups revealed that the SF^110^ α-irradiated fibroblasts exhibited alterations in the methylome profile of 2.9% of genes relative to Horvath’s DNAm clock and 2.0% relative to the age-associated genes from the PhenoAge clock (Fig. 3a). In the MF treatment groups, the MF^23^, MF^1100^, and MF^2200^ irradiations induced DNAm changes in 0.58%, 6.1%, and 17% of genes, respectively, compared to the genes associated with Horvath’s DNAm clock. Similarly, compared to the genes from the PhenoAge DNAm clock, MF^23^, MF^1100^, and MF^2200^ affected 0.59%, 5.0%, and 13% of genes, respectively (Fig. 3a). These findings demonstrated that MF irradiations induced alterations in the DNAm patterns of a greater number of genes associated with DNAm biomarkers of aging compared to SF exposures. Although we observed a clear trend for a dose-dependent increase in these alterations, enrichment analysis revealed that these findings relative to the two DNAm clocks are not significantly represented (hypergeometric *p* value > 0.05) (Fig. 3b). For a comprehensive list of the affected regions in each irradiated group, refer to Additional file 1: Table S4. To further elucidate the biological implications, we next conducted an enrichment analysis using Ingenuity Pathway Analysis (IPA) with the gene lists affected by the aging-associated DMRs identified in the SF- and MF-treated groups. The analysis revealed that the top five most significantly enriched canonical pathways (Fig. 3c) were associated with cell death and survival, cellular development, cell cycle functions, cell-to-cell signaling interactions, and cell morphology. Furthermore, functional analysis indicated that these genes were linked to cancer and mitochondrial dysfunction, events that have been described as the molecular pillars of aging [47, 48].

## Possible transitory defense mechanism of the fibroblasts against carcinogenesis to the MF α-irradiation

**Fig. 4:**
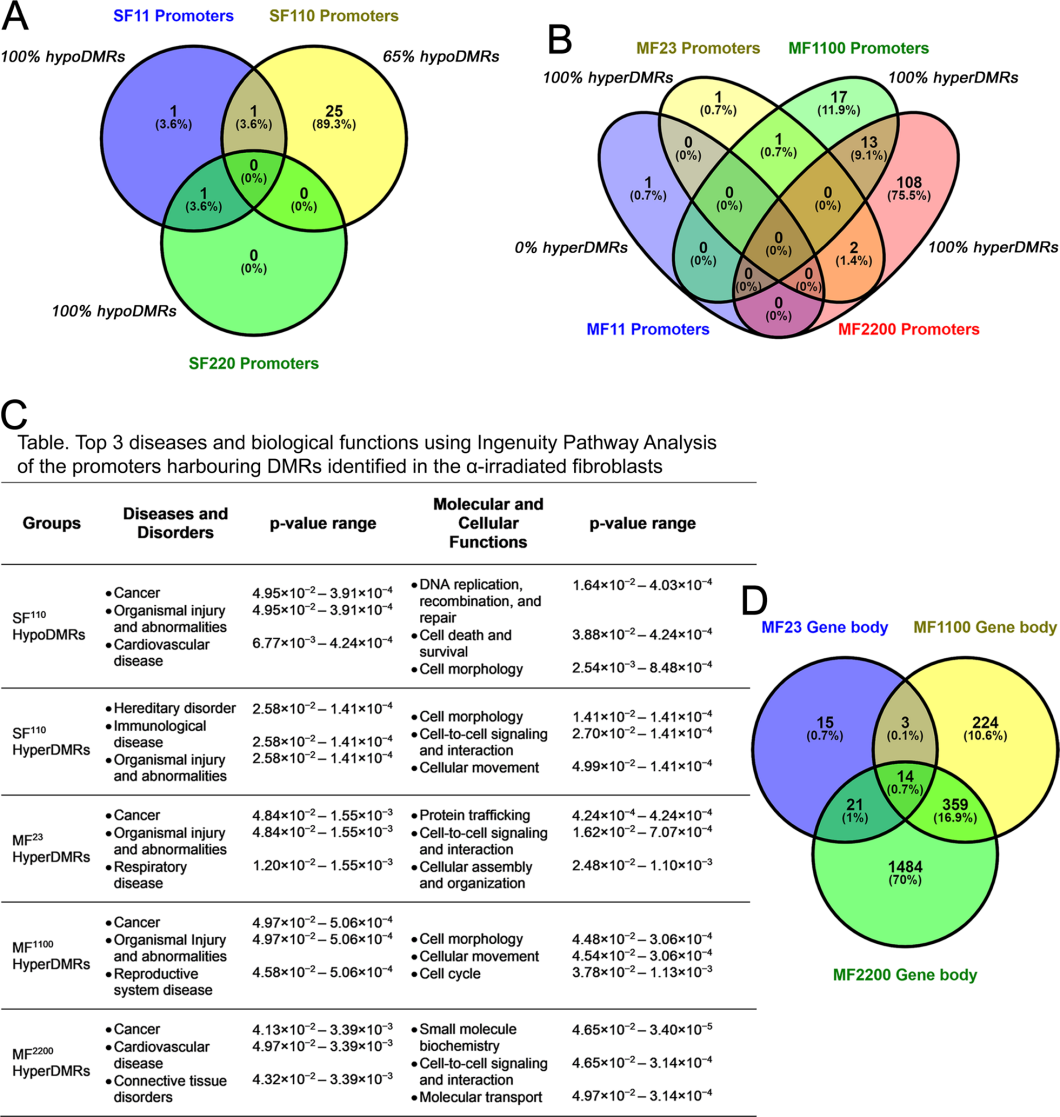
Promoters and gene body regions whose methylome was altered by α-irradiation in lung fibroblasts. **A** Number of promoters that were either hypo or hypermethylated following single-fraction (SF) α-irradiation. **B** Number of promoters that were either hypo or hypermethylated following multi-fraction (MF) α-irradiation. **C** Table displaying the top diseases and biological functions associated with the affected promoters in the α-irradiated fibroblasts characterized using QIAGEN Ingenuity Pathway Analysis. **D** Number of gene body regions that were hypermethylated following MF α-irradiation. (Venn diagrams created using Oliveros [63])

We then investigated whether established or novel epigenetic biomarkers of lung carcinogenesis could be identified in the α-irradiated fibroblasts. Alteration in the 5mC levels at gene promoters is a key event in tumorigenesis. In the SF groups, we identified 30 DMRs across the SF^11^, SF^110^, and SF^220^, which induced DNAm changes in 3, 26, and 1 promoters, respectively (Fig. 4a). Among these DMRs, 17% were hypomethylated and associated with oncogenesis, specifically NAD(P)-dependent steroid dehydrogenase-like (*NSDHL*), solute carrier family 16 member 3 (*SLC16A3*), copine 1 (*CPNE1*), LIF receptor subunit alpha (*LIFR*), and protein kinase CAMP-activated catalytic subunit gamma (*PRKACG*). The list of all promoters affected by the SF irradiation can be found in Additional file 1: Table S5. Conversely to the few DMRs within promoter regions detected in the SF doses, the MF irradiations resulted in 164 DMRs located within these regions. More specifically, MF^11^, MF^23^, MF^1100^, and MF^2200^ were the doses that altered the DNAm levels of 1, 4, 31, and 123 promoters, respectively (Fig. 4b). Unlike the SF exposures, increased 5mC sites were observed in all promoters from the MF groups, except for 2 hypoDMRs identified in the MF^11^ and MF^2200^ treatments.

To identify DNAm markers of α-induced tumorigenesis, we then examined promoters that were commonly altered by multiple radiation doses. Thirteen promoters were hypermethylated by both MF^1100^ and MF^2200^, six of which were linked to oncogenic activities. The genes regulated by these six promoters include calcium/calmodulin-dependent protein kinase II alpha (*CAMK2A*), long intergenic non-protein coding RNA 884 (*LINC00884*), long intergenic non-protein coding RNA 1624 (*LINC01624*), melanoma-associated antigen A12 (*MAGEA12*), phosphoribosyl pyrophosphate synthetase 2 (*PRPS2*), and zinc finger DHHC-type palmitoyltransferase 16 (*ZDHHC16*). The expression of *CAMK2A* has been shown to support tumor-initiating cells in lung adenocarcinomas [50]. Long non-coding RNAs, including *LINC00884* and *LINC01624* have emerged as critical epigenetic regulators in various cellular functions and human diseases such as cancer and aging [51]. Up-regulation of different members of the MAGE-A gene family has been documented in various types of cancers [52], and in the case of *MAGEA12*, its role is to repress the expression of tumor suppressor genes [53]. *PRPS2* is involved in promoting increased nucleotide biosynthesis [54]. The expression of *ZDHHC16* is involved in the early stages of DNA damage response [55], which can be beneficial in some situations, particularly during exposure to ionizing radiation, an event known to induce mutations in the DNA of the irradiated cells. However, *ZDHHC16* has also been associated with oncogenic events [56].

In addition to these promoters, we identified two other promoters hypermethylated by MF^23^ and MF^2200^, the promoters of the *ras* homolog family member T2 (*RHOT2*) and the long intergenic non-protein coding RNA 2280 (*LINC02280*) genes. The FES proto-oncogene (*FES*) promoter was also hypermethylated by two radiation doses, MF^23^ and MF^700^. For the list of all promoters affected by the MF α-irradiation doses, refer to Additional file 1: Table S6.

Functional classification using IPA revealed that the most prominent disease and disorder associated with the genes affected by the differential promoter hypermethylation triggered by α-irradiation to MF^23^ and MF^1100^ was cancer followed by organismal injury and abnormalities. As for the highest MF dose, MF^2200^, the top disease associated with the promoters harboring hyperDMRs was also cancer followed by cardiovascular diseases (Fig. 4c). Similarly, the genes affected by the differential promoter hypomethylation induced by the SF^110^ were also associated with cancer, organismal injury and abnormalities, and cardiovascular disease. However, the hypermethylated promoters from the SF^110^ were involved in hereditary disorders and immunological diseases (Fig. 4c).

Cancer is also characterized by hypermethylation in the body region of oncogenes, leading to increased gene expression, and hypomethylation in the body region of tumor suppressor genes, resulting in decreased gene expression [57]. Within the SF groups, we found a total of 474 DMRs localized within this region, of which only 4.6% were hypermethylated. The hyperDMRs were observed in response to SF^110^, potentially affecting 21 genes. In contrast, the MF irradiations led to 3,487 DMRs within the gene body region, of which 98.5% exhibited increased 5mC levels. The MF doses that induced an elevation in 5mC sites at the body region were MF^11^, MF^23^, MF^1100^, and MF^2200^ with 1, 55, 678, and 2,701 hyperDMRs, potentially affecting 1, 53, 600, and 1,878 genes, respectively.

Fourteen genes were found to be commonly hypermethylated at their body region by three MF doses, MF^23^, MF^1100^, and MF^2200^ (Fig. 4d). Functional analysis of these 14 genes with IPA revealed that 11 were involved in carcinogenesis. These 11 genes include acrosin binding protein (*ACRBP*), UDP-GalNAc: polypeptide N-acetylgalactosaminyltransferase 9 (*GALNT9*), MAGUK p55 scaffold protein 2 (*MPP2*), *FES*, ecotropic viral integration site 5-like (*EVI5L*), ATPase phospholipid transporting 8b3 (*ATP8B3*), ATP-binding cassette subfamily A member 7 (*ABCA7*), basal cell adhesion molecule (*BCAM*), rho guanine nucleotide exchange factor 16 (*ARHGEF16*), phospholipase C eta 2 (*PLCH2*), and Janus kinase 2 (*JAK2*). The *GALNT9* gene was found to be both an anti-tumor suppressor and possesses oncogenic capacities, depending on the type of cancer [58]. The function of the other four genes *JAK2*, *EVI5L*, *ABCA7*, and *ARHGEF16* is not clear; however, they have been reported as genes with potential oncogenic activities [59–62]. Furthermore, four of these genes including *JAK2*, *FES*, *PLCH2*, and *ARHGEF16* were also found to be involved in immunological response and cellular signaling. Data analysis using the complete list of genes whose gene body regions were hypermethylated by the MF doses uncovered that the top disease and disorder associated with these genes was also cancer.

Interestingly, although carcinogenesis-related events were primarily observed in the MF groups, our data revealed that α-irradiation to SF^110^ decreased the DNAm levels within the body region of O^6^-methylguanine-DNA methyltransferase (*MGMT*). The expression of *MGMT* is known to be inhibited in lung cancer. Another gene whose 5mC levels were altered at the body region by multiple α-irradiation doses including SF and MF exposures was RB transcriptional corepressor 1 (*RB1*). The effects on the DNAm levels of *RB1* were dependent on the dose fractionation, where SF^110^ resulted in hypoDMRs, and irradiations to MF^1100^ and MF^2200^ led to hyperDMRs within the *RB1* gene. The PANTHER classification system revealed that the RB1 protein is associated with the regulation of cellular and developmental processes. RB1 is considered a tumor suppressor protein.

## Mitochondria-related DNAm biomarkers of α-radiation are under-represented

Mitochondria are dynamic organelles located in the cytoplasm of eukaryotic cells that function as the main source of cellular energy and are known to play a pivotal role in the epigenetic effects induced by ionizing radiation [64]. Consequently, we investigated DNAm biomarkers that can potentially alter mitochondrial structure and function in the α-irradiated fibroblasts.

Eukaryotic mitochondria contain a circular genome of 16,569 basepair (bp), which encodes 37 genes [65]. Only in the last two decades, it was uncovered that the expression of these mitochondrial genes could be regulated by DNAm, although to a lesser extent than the nuclear genes [65, 66]. Despite the potential for mitochondrial DNA to be methylated, we did not detect any alteration in 5mc levels in the mitochondrial DNA. However, we identified changes in the DNAm patterns of nuclear-encoded mitochondrial genes. These genes, encoded by nuclear DNA, are required for normal mitochondrial function. The ratio of nuclear-encoded mitochondrial genes to the total number of nuclear genes is relatively low with an estimated 1,650 in 20,000 (~ 8.3%) genes in humans [67, 68].

**Fig. 5:**
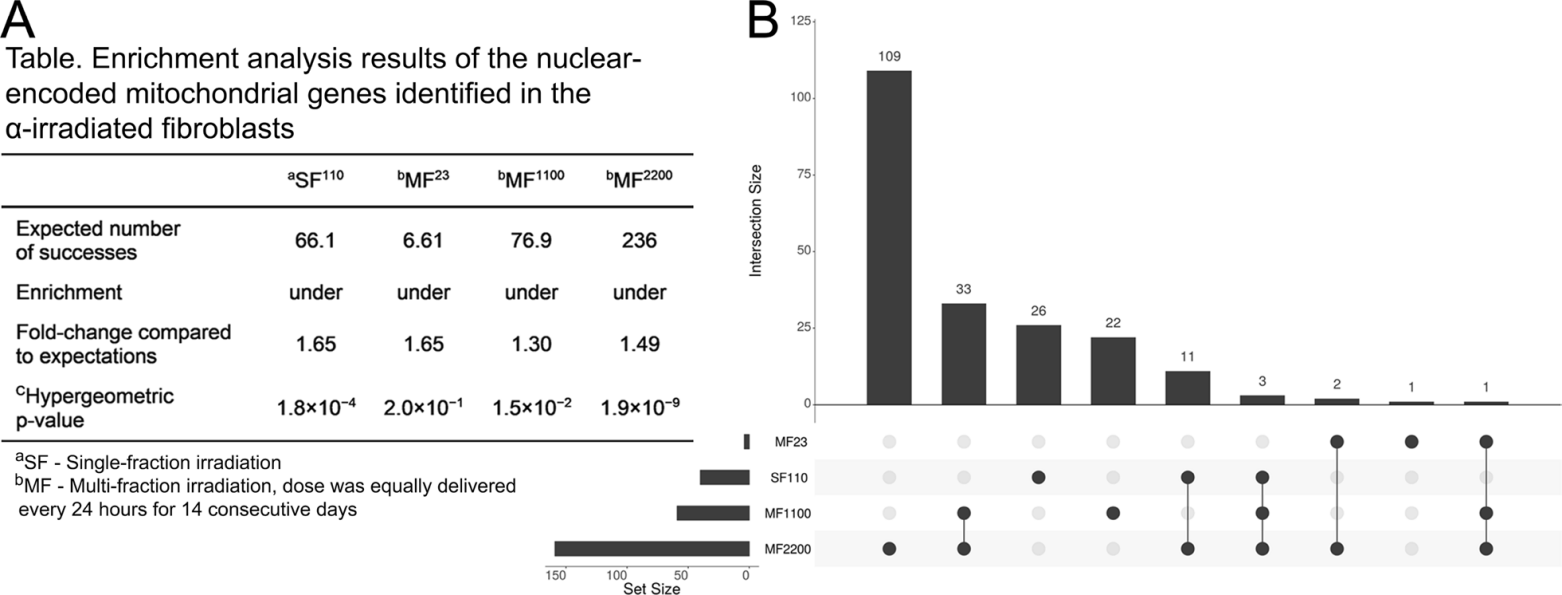
Nuclear-encoded mitochondrial genes identified in the fibroblasts following irradiations to α-particles. **A** Table displaying the results from the enrichment analysis of the nuclear-encoded mitochondrial genes whose methylation levels were altered in the α-irradiated fibroblasts, computed using the cumulative distribution function of the hypergeometric equation [49]. **B** UpSet plot exhibiting the number of nuclear-encoded mitochondrial genes harboring DMRs identified in the fibroblasts across the different α-irradiation doses

Using the 1,655 nuclear-encoded mitochondrial gene list provided by Elsadany et al. [68], we identified 40, 4, 59, and 159 of these genes harboring DMRs in the fibroblasts α-irradiated to SF^110^, MF^23^, MF^1100^, and MF^2200^, respectively. Further enrichment analysis using the cumulative distribution function of the hypergeometric equation [49] revealed that the nuclear-encoded mitochondrial genes detected in the SF^110^, MF^1100^, and MF^2200^ irradiated cells were significantly enriched with a cumulative hypergeometric p value < 0.05 (Fig. 5a). However, these genes were found to be under-represented.

We then examined the genes associated with mitochondrial dysfunction whose DNAm levels were commonly altered by more than one radiation dose (Fig. 5b). The gene with the most alterations was *RHOT2*, and it encodes for trafficking proteins involved in mitochondria homeostasis and apoptosis [69]. HyperDMRs were identified in the gene body region of *RHOT2* following α-irradiation to MF^1100^ and MF^2200^. Additionally, its promoter was hypermethylated by MF^23^ and MF^2200^ treatments. Furthermore, we identified 11 nuclear-encoded mitochondrial genes whose DNAm patterns were altered by both the SF^110^ and the MF^2200^ treatments. However, SF^110^ led to reduced 5mC sites within the nuclear-encoded mitochondrial genes, whereas MF^2200^ resulted in increased DNAm levels within the same genes.

## Discussion

In this study, we investigated the effects of human-relevant doses of α-radiation and the comparison of SF and MF exposures on the methylome profile of a normal human embryonic lung fibroblast cell line. There is evidence that ionizing radiation can be a potent epigenotoxic agent [13]. Nevertheless, existing knowledge on the distribution of 5mC levels in the lung cells following α-irradiation is limited. Our findings revealed that in vitro α-irradiation significantly impacts the methylome of the lung fibroblasts, with the effects being highly dependent on the dose, and the exposure regimen (SF versus MF irradiation). More specifically, when the total dose was delivered in a SF treatment, it either had no effect on the methylome or led to decreased DNAm levels in the irradiated cells relative to the control. In contrast, when the total dose was delivered as MF with one fraction given every 24 h over a period of 14 days, it primarily induced a gain in 5mC sites compared with the non-irradiated cells. This is important, as repetitive (often cyclical) exposure to α-radiation is the exposure modality that humans most commonly experience due to residential ^222^Rn exposure [1–7]. Moreover, at equivalent SF doses, the MF α-irradiation resulted in DNAm changes of numerous genes linked to events associated with lung carcinogenesis, cardiovascular disease, and aging. Alterations in methyl levels of key genes linked to mitochondrial damage, a biomarker of ionizing radiation, were also detected primarily in the MF α-irradiated fibroblasts.

In the few available studies reporting on the effects of radiations of high linear energy transfer (LET, a measure of ionizing track density) on DNAm levels, which includes α-particles, different radiation exposure units were used, rendering it challenging to compare results across studies. Therefore, to address this issue, we introduced a standardized measure called “*our dose estimate*” whenever possible. It is important to highlight that *our dose estimate* is not an accurate representation of the exposure dose but an estimate. In studies where ^222^Rn levels were reported in Bq·m^−3^, we derived *our dose estimate* using the effective dose equation proposed by the International Commission on Radiological Protection (effective dose mSv = ^222^Rn level Bq·m^−3^ × exposure time h × dose coefficient mSv per mJ·h·m^−3^). We assumed a standard equilibrium factor of 0.4, which is defined as the concentration difference between the ^222^Rn gas and its solid progeny. Alternatively, if the study reported absorbed radiation doses in Gy, we calculated *our dose estimate* in Sv using a radiation weighting factor of 20 for α-particles and, if necessary, 0.12 as the radiation weighting factor for lung exposures [70]. For reference, the effective doses of our cells that were α-irradiated to SF and MF doses of 2.0, 11, 23, 110, 220, 1,100, and 2,200 in mGy are in the order of 4.8, 26, 55, 260, 530, 2,600, and 5,300 in mSv, respectively.

Our in vitro results of lower 5mC levels identified in the SF-irradiated fibroblasts compared with the unexposed cells are consistent with a previous α-radiation study conducted by Huang et al. [31]. The authors in this study observed a reduction of 38 to 90% in DNAm levels after exposing human bronchial epithelial BEAS-2B cells to 20,000 Bq·m^−3 222^Rn for 30 min. Consequently, Huang and colleagues detected a decrease in DNAm in the irradiated cells with a SF low *dose estimate* of 0.067 mSv. Unlike Huang et al. [31], we did not detect any alterations in the methylome landscape at the lowest SF doses, despite similar exposure levels. This difference could be attributed to various factors, including the type of lung cells used in each study, the duration of exposure, the irradiation rate, and the incubation period following irradiation. In the study by Huang et al. [31], it was unclear how long the irradiated cells were incubated prior to collection. Nevertheless, it is apparent that SF irradiation to α-particles reduces 5mC levels in lung cells.

High DNAm levels within the promoter region of *MGMT*, a tumor suppressor gene, have been observed in response to high LET α-exposure [12] and in lung tumors [71]. The *MGMT* gene encodes a DNA repair enzyme responsible for cellular defense against the mutagenic and toxic effects of alkylating agents. Here, we detected a decrease in 5mC levels at the body region of the *MGMT* gene in the fibroblasts α-irradiated to SF^110^ compared with the control cells. It is important to note that the DNAm levels at either the promoter site or the gene body region can regulate the transcriptional patterns of a gene. This was confirmed in a study by Moen and colleagues where they demonstrated that the expression of *MGMT* can be reduced either through promoter hypermethylation or gene body hypomethylation [72]. The increase in the DNAm rates of the *MGMT* promoter was observed in the sputum of Chinese uranium miners who were exposed to significant levels of ^222^Rn [12]. The sputum was collected from 91 male miners aged between 42 and 55 years old exposed to ^222^Rn for 13 to 35 years of service. Interestingly, it has been shown by Ginno et al*.* [73] that the DNAm rate in the gene body region is higher compared with that of the promoter sites. Therefore, the lack of methylation changes in the promoter region of the *MGMT* gene observed in our α-irradiated fibroblasts is not surprising as the methylome from our SF-irradiated cells had only 1 h to undergo any changes in response to the irradiation as opposed to several years as in the study by Su et al. [12]. Despite the different genomic regions from the *MGMT* gene between our study and that of Su and colleagues in which 5mC sites were altered following the α-irradiation, the resulting *MGMT* expression levels are likely to be reduced in both studies.

DNA methylation levels within the promoter region of the *MGMT* gene were also assessed by Belinsky et al. [11]. However, the authors were not able to reproduce the findings of Su and colleagues [12], as no changes in DNAm from miners exposed for 24 years to the α-emitter plutonium-239 at the Russian nuclear enterprise MAYAK were detected in the *MGMT* promoter of lung adenocarcinomas. The adenocarcinomas were collected from 71 MAYAK employees (63 males and 8 females), and 69 non-worker male controls. During their time at the Russian nuclear plant, the MAYAK workers were exposed to a wide range of mixed low LET gamma radiation and high LET α-particle exposure doses with a median level of 1.3 and 0.52 Gy, respectively. *Our dose estimate* of the α-radiation from the plutonium-239 that the MAYAK workers were exposed to was of the order of 1,250 mSv. Changes in DNAm elicited by high LET radiation appear to be highly dependent on the dose and the incubation time post-irradiation. Such findings were reported in a study by Lima et al. [25] in which the authors found different DNAm profiles in six genes including *MGMT*, in the lungs of mice in vivo irradiated to 100, 300, and 1,000 mGy of high LET ion-particles and then assessed at 1, 7, 30 and 120 days after total body irradiation. The results by Lima and colleagues may explain the lack of changes in the *MGMT* gene in our MF-irradiated fibroblasts as they reported no significant alterations in the DNAm of the *MGMT* promoter in the lungs after 30 days post-radiation. However, there was a significant increase in 5mC levels in the *MGMT* promoter in the lungs in vivo irradiated to 100 mGy and assessed 1-d post-exposure, whereas after 7 and 120 days post-radiation, the methyl levels were significantly decreased in the lungs irradiated to 100 and 300 mGy. Taken together, these findings suggest that aberrant DNAm of the *MGMT* gene could be a suitable epigenetic marker of high LET α-radiation exposure; however, the promoter concomitantly with the body region of the *MGMT* gene should be considered in the DNAm assessment and at least two time points, shortly after and over an extended period following the irradiation.

Both promoter hypermethylation leading to a reduction in tumor suppression activity and promoter hypomethylation resulting in an increased expression of oncogenes are considered hallmark events in a variety of cancers including radiation-induced lung carcinogenesis [13, 29]. Here, we identified six genes associated with tumorigenesis whose promoters were commonly hypermethylated by α-irradiation to MF^1100^ and MF^2200^. However, surprisingly for our MF irradiations, these genes have been shown to indirectly facilitate oncogenic signals. It is unclear as to the reason underlying the promoter hypermethylation of these oncogenes observed in our MF-irradiated cells. Nevertheless, our findings suggest that the MF irradiations conditioned the fibroblasts to respond to the exposure by promoting an adaptive or defensive response to the stressor, a mechanism commonly detected in cells exposed to other environmental insults [74].

Decreased tumor suppression activity by gene body hypomethylation and increased expression of oncogenes by gene body hypermethylation have become important signature events in carcinogenesis [75]. Our results showed that the vast majority of DNAm changes detected in the body site of genes associated with cancer were induced by the MF rather than SF radiation exposures. Following the irradiations, we uncovered fourteen genes involved in tumorigenesis whose body regions were commonly hypermethylated by α-irradiation to MF^23^, MF^1100^ and MF^2200^. Contrarily to the promoters, the genes identified in our study harboring hypermethylated body regions have been reported to indirectly facilitate anticancer activities. However, in some cases, their role is not fully understood. Another gene associated with cancer events whose 5mC levels were commonly increased at the gene body region by MF irradiations, MF^1100^ and MF^2200^, was *RB1*. The protein encoded by *RB1* is a key regulator of the cell cycle entry, and therefore, it is considered a potent tumor suppressor [76]. Strikingly, the physiological response of defending against rather than succumbing to the MF irradiation is consistent in both the hypermethylated promoters and hypermethylated gene body regions.

Aside from cancer and organismal injury and abnormalities, cardiovascular disease was one of the top three diseases and disorders linked to the hypermethylated promoters in our highest MF dose and to the hypomethylated promoters from our medium SF dose. An excess incidence of cardiovascular disease has been reported among atomic bomb survivors, astronauts, and patients treated with radiotherapy; all groups exposed to significant doses of ionizing radiation [77–79]. The association between ionizing radiation and its deleterious effects on the heart and blood vessels was challenging to establish as these effects appear years to decades after the exposure [80]. Here, we identified seven genes immediately post-irradiation associated with cardiovascular diseases whose 5mC levels were altered by α-radiation at their promoter region. These genes were crystallin alpha B (*CRYAB*), coagulation factor II thrombin receptor (*F2R*), prostaglandin D2 synthase (*PTGDS*), sodium voltage-gated channel alpha subunit 4 (*SCN4A*), FA complementation group G (*FANCG*), and two microRNAs. Accordingly, future human studies are warranted to confirm the involvement of epigenetic changes immediately post-irradiation as a biomarker of potential latent cardiotoxicity.

Since the early twentieth century, the detrimental effects of ionizing radiation have been attributed largely to nuclear DNA damage [81]. However, in the last decades, it has been demonstrated that the molecular outcomes of exposure to ionizing radiation are highly reliant on the strong functional performance of mitochondria [64, 82]. Since mitochondria are one of the major cytoplasmic targets for ionizing radiation, it may seem contradictory that we did not identify any alteration in 5mC levels on the mitochondrial DNA of the α-irradiated fibroblasts. However, because of the very low percentage of mitochondrial DNA content (~ 0.25% of the total cellular DNA) relative to the nuclear DNA, it is difficult to significantly detect any methyl changes in the mitochondrial DNA when performing a genome-wide study [83, 84]. As for the nuclear-encoded mitochondrial genes, albeit their low percentage relative to the total nuclear genes (~ 8.3%), we identified several DMRs within regions of these genes in three MF irradiation groups. Specifically, MF^23^, MF^1100^, and MF^2200^ induced 5mC aberrations in ~ 5.1%, ~ 6.4% and ~ 5.6% of nuclear-encoded mitochondrial genes, respectively, relative to the total nuclear genes detected in the α-irradiated cells. The hypergeometric distribution analysis revealed that the percentages of the identified nuclear-encoded mitochondrial genes were significantly under-represented, suggesting that the mitochondria prefer other more rapid methods of adapting to the radiation-induced oxidative stress such as cysteine-mediated post-translation of proteins [85]. Regardless, it would be interesting to investigate whether the DNAm changes in the under-represented nuclear-encoded mitochondrial genes have an impact on the mitochondrial defense mechanism against radiation-induced oxidative stress. This is especially important since hypermethylation at the gene body region of glutathione peroxidase 4 (*GPX4*) was detected in our highest MF α-radiation dose, potentially increasing its expression. The protein encoded by this gene is a key enzyme involved in protecting the cells from damage caused by reactive oxygen species and xenobiotics [86].

DNA methylation clocks have been proposed as the most promising molecular marker for predicting life expectancy in humans [87]. Even though there is no conclusive evidence that the α-particles altered the epigenetic age of our irradiated cells, we observed a non-significant trend for a dose-dependent increase in the number of genes associated with aging whose DNAm levels were mainly hypermethylated as a result of the MF exposures. Established epigenetic clocks, algorithms designed to estimate the epigenetic age of a tissue, are constructed based on predefined probes imprinted in Illumina DNAm microarray chips, resulting in limited coverage of the epigenome. In contrast, our study uses MeDIP-seq to identify DNAm changes in α-irradiated lung fibroblasts, capturing all potential alterations in DNAm in response to α-radiation, and likely some of these changes are not represented in the Illumina chips. Taken together, these findings indicate that high LET α-radiation has the potential to alter the epigenetic age in the irradiated fibroblasts; however, this hypothesis needs to be further tested.

It is important to note that because of some experimental caveats, the effects on the methylome identified from the SF irradiations may require additional validation. The difference in time periods from the initial exposure to the harvest time between the SF and MF irradiations is significant. The cells from the SF regimen were collected within 1 h post-irradiation, whereas the cells from the MF exposures were harvested 14 days from the first fraction of α-irradiation. The 1 h incubation period post-irradiation that the SF cells were subjected to may have not been sufficient to induce alterations in the methylome patterns in response to the applied radiation. Therefore, validation of the results from the SF irradiations is warranted. The premise behind this observation is the study by Yamagata and colleagues [88], where the authors investigated the turnover of DNAm in human cells and found that after 2 h there was a significant de novo DNAm, albeit at a level one order of magnitude less than that of DNAm after 48 h. Additionally, the WI-38 cell line used in this study was derived from a 3-month-old female; therefore, careful consideration must be given when extrapolating these results to adult tissue, as embryonic cells are known to have different methylome profiling compared with adult cells [89]. Consequently, the response of embryonic cells to radiation may differ from that of the established adult cells.

In conclusion, our findings reveal that both SF and MF α-exposures induce changes in the methylome of lung fibroblasts, with a dose-dependent effect observed in the MF irradiations. Specifically, SF exposure primarily led to a reduction in DNAm, whereas MF irradiation mainly elicited the opposite effect. These data provide a direct comparison of the effects of SF and MF irradiation on 5mC levels in response to a range of human-relevant doses of α-particles, supporting the use of both exposure regimens for examining the effects of ionizing radiation on humans. Although the contrasting effects on the methylome in response to the SF as opposed to the MF irradiation were clear, these findings remain to be confirmed, as the SF time post-irradiation was likely insufficient for the methylome to fully respond to the high LET radiation. However, in general, the DNAm data are of note as they expand the significant genes and their regions exhibiting the influence of human-relevant doses of α-radiation on the methylome of lung cells. Since humans are repetitively exposed to cycles of high and low doses of α-particles as they move between residential ^222^Rn levels, and lower levels in vehicles, outside, and in occupational and retail settings, which occur at a wide range of doses and dose rates [4, 5, 7, 90], conducting studies with a broad range of ionizing radiation doses is important to better understand its effects in all exposure settings. In addition, our findings shed some light on the potential epigenetic modifications associated with cardiovascular diseases triggered by α-radiation. The effects of MF α-irradiation, as opposed to SF, were also captured in the DNAm of mitochondria-related genes, which could potentially lead to mitochondrial dysfunction. However, the number of mitochondria-associated genes harboring DMRs identified in the MF α-irradiated cells was under-represented, suggesting that adaptive mechanisms other than DNAm may play a greater role in radiation-induced oxidative stress in mitochondria. Moreover, a high percentage of the genes affected by the nuclear DNAm changes in our α-irradiated fibroblasts were also linked to aging and carcinogenesis. A dose-dependent trend on the number of genes affected by the aging-associated DMRs was observed in our MF-irradiated cells, indicating that α-radiation has the potential to alter the epigenetic age of the cells. While a greater number of key genes associated with cancer development were detected in the cells following MF irradiations compared with SF treatments, the outcome of this alteration seems to be a protective mechanism of the cells in response to the applied stressor. However, further investigation is warranted to determine whether the DNAm biomarkers observed following the MF irradiations, indicating a defensive response of the fibroblasts to the exposure, persist over a longer exposure period. Given the strong correlation between prolonged exposure to high LET α-radiation and carcinogenesis, as well as other disorders, it is important to investigate the dose and dose duration required for this cellular protective mechanism to be overcome by continuous α-radiation exposures. Lastly, it would be valuable to assess whether an extended post-irradiation time following SF exposure confirms the different DNAm profiles observed in this study for these types of exposures.

## Methods

### Cell culture

Human WI-38 cells, a normal fibroblast cell line derived from a 3-month-old female lung tissue, were commercially acquired from the American Type Culture Collection (ATCC #CCL-75). Cell cultures were maintained as described by Stanley et al*.* [91]. Briefly, WI-38 cells were propagated in EMEM medium (ATCC #30–2003) supplemented with 10% fetal bovine serum (Gibco #12,483–020), 50 U/mL penicillin, 50 µg/mL streptomycin (Gibco #15,070–063), and 2 mM L-glutamine (Gibco #25,030–081). All cultures were incubated at 37 °C with 5% CO_2_ in a humidified atmosphere. Cell lines were regularly tested for mycoplasma contamination.

## Particle irradiation

Cells were plated in 96-well polystyrene plates (Sarstedt #83.3924) in a total of 216 wells per treatment per replicate at a concentration of 1.5 × 10^5^ cells·mL^−1^, and allowed to adhere for 8 h. Cells were then irradiated with either SF or MF treatments of α-particles at room temperature as described by Stanley et al. [91].

Cell irradiation was performed by first removing the media from the wells, and subsequently inserting an americium-241 source holder into the corresponding wells. Upon delivering the desired dose, the sources were removed, and the media was replaced. The distance between the α-particle sources and the sample surface varied depending on the administered dose, either 1.8 or 8.8 mm. Cells were subjected to seven doses of α-particles, each dose was irradiated using two exposure regimens: a SF regimen, where the total dose was delivered at once, and a MF approach, where the total dose was equally distributed into 14 fractions, with one fraction delivered every 24 h. Dose rates are described in Additional file 1: Table S1 for the SF treatments and Additional file 1: Table S2 for the MF irradiations. The irradiation per dose for each regimen was conducted in 4 biological replicates with 216 wells pooled per replicate. Each replicate was independently irradiated and assessed. For the SF irradiations, if the time to deliver the total dose exceeded 2 min, the total dose was delivered in fractions of 2 min with media replacement and 5 min incubation in between fractions.

The SF α-irradiation doses used were 2.0, 11, 23, 110, 220, 1,100, and 2,200 mGy, whereas the MF irradiation doses were 0.14, 0.79, 1.6, 7.9, 16, 79, and 160 mGy per day. Sham controls were included for each dose and exposure regimen. For more information on the doses delivered, refer to Additional file 1: Table S1 and Additional file 1: Table S2 for SF and MF irradiations, respectively. The high throughput α-particle irradiator system and protocol used in this study were extensively validated and optimized with a wide range of α-particle doses on human and yeast cells, demonstrating that these doses are below lethality thresholds [91]. The authors reported a dose-dependent 20 to 40% decrease in viability in human cells only between the doses of 2,800 and 11,000 mGy following 24 h post-α-irradiation.

Cells in both SF and MF exposure regimens were trypsinized and collected after the last irradiation following 1 h incubation at 37 °C in a 5% CO_2_ humidified atmosphere. Collected cells were immediately flash-frozen in liquid nitrogen and subsequently stored at − 80 °C until total DNA was extracted.

## DNA Extraction

Total DNA was extracted from frozen WI-38 cell pellets from both SF and MF α-irradiated groups using a QIAamp DNA Mini Kit (QIAGEN, Germany) according to the manufacturer’s protocol with minor modifications. Cell pellets were resuspended in phosphate-buffered saline solution and treated with proteinase K and RNase A. The DNA-loaded spin columns were washed three times with buffer AW2 to ensure the complete removal of salts. DNA was eluted in Tris–EDTA (TE) buffer (low EDTA, pH 8.0) after 5 m of incubation at room temperature. The quality and concentration of the DNA were determined spectrophotometrically using a NanoPhotometer NP80 (Implen, Germany). The total amount of DNA obtained was at least 7 μg. DNA samples displayed A260/A280 ratios in the range of 1.8 to 2.0 and A260/A230 ratios higher than 2.1.

## MeDIP-seq

Extracted intact DNA (5 μg in 130 μL TE buffer, low EDTA, pH 8.0) was randomly sheared into fragments with an average size of 200 bp using an S220 focused-ultrasonicator (Covaris, USA). The sonication was performed at 7 °C for 180 s with a 10% duty factor, peak incident power set at 175 W and cycle per burst set at 200. To prevent DNA loss, DNA LoBind tubes (Eppendorf, Canada) were used throughout the experiment. End-repair, adaptor ligation, and size selection were subsequently performed on the fragmented DNA (1.2 μg) using a NEBNext Ultra II DNA library prep kit for Illumina (New England Biolabs, Canada) following the manufacturer’s guidelines. The resulting adaptor-ligated DNA underwent immunoprecipitation (IP) with a 5-mC antibody using a MagMeDIP kit (Diagenode, USA) as described in the manufacturer’s protocol with the modification that the starting volume of the adaptor-ligated DNA for the IP incubation was 15 µL (volume of water was adjusted). A portion of the MeDIP mix was reserved as input; this fraction did not undergo immunoprecipitation. The immunoprecipitated adaptor-ligated DNA and input fraction were further purified with an IPure kit v2 according to the manufacturer’s instructions (Diagenode, USA).

The recovery and enrichment of the methylated DNA were assessed by quantitative polymerase chain reaction (qPCR) using primer sets specific for the spike-in controls (Diagenode, USA) as well as for the endogenous hypermethylated promoter region of testis-specific histone 2B (Diagenode, USA), and for the endogenous hypomethylated transcriptional start site of glyceraldehyde 3-phosphate dehydrogenase (Diagenode, USA). The qPCR thermal cycling was conducted with a QuantStudio 3 Real-Time PCR System (Applied Biosystems, USA) using a PowerUp SYBR green master mix (Applied Biosystems, USA) with the following cycling profile: 2 min Uracil-DNA glycosylase activation at 50 °C, 2 min dual-lock DNA polymerase at 95 °C, 40 cycles of 95 °C for 15 s, and 60 °C for 1 min. A dissociation step was performed at the end of each qPCR run between 60 and 95 °C with 0.1 °C increments. The average recovery rate of methylated DNA fragments was found to be 47%, and that of unmethylated fragments was lower than 0.2%. The fold-enrichment ratio between methylated and unmethylated fragments is recommended to exceed 25 [92]. Our libraries displayed an average enrichment of 3,629-fold.

Thereafter, the libraries from the purified input fraction and the immunoprecipitated adaptor-ligated DNA were linked to unique dual index primers (New England Biolabs, Canada) and amplified by PCR using a NEBNext Ultra II DNA library prep kit for Illumina (New England Biolabs, Canada) as described in the manufacturer’s guidelines. The number of cycles used for the input fraction and the MeDIP samples was 7 and 8, respectively. The PCR products were further purified using NEBNext sample purification beads (New England Biolabs, Canada). Libraries were quantified using a KAPA library quantification kit (Kapa Biosystems, USA) with a QuantStudio 3 Real-Time PCR System (Applied Biosystems, USA). Libraries were pooled at equimolar concentrations and sent to Génome Québec (Montréal, Canada) where they were subjected to paired-end sequencing with a 150 bp read length using an Illumina NovaSeq 6000 S4 platform (Illumina), generating around 138—226 million raw reads per library.

## Sequencing data processing

An initial quality control to screen for contaminants was conducted on the paired-end sequenced raw reads using FastQ Screen software (v0.4.1) [93]. The paired-end reads were then analyzed with FastQC (v0.11.9) (https://www.bioinformatics.babraham.ac.uk/projects/fastqc/) for general read quality assessment. The reads were further processed with TrimGalore (v0.6.6) (https://www.bioinformatics.babraham.ac.uk/projects/trim_galore/) to both remove adaptors and filter low-quality reads. The parameters that were used with TrimGalore were: trimming of 1 bp at the 5’ and 3’ end for read 1 and 2 (–clip_R1 1, –clip_R2 1, –three_prime_clip_R1 1, and –three_prime_clip_R2 1), removal of unknown base pairs from both ends (–trim-n), filtering reads with length < 50 bp and quality score < 20 on the Phred scale (–length 50 and -q 20), and removal of adaptors with an overlap stringency of 5 bp (–stringency 5). The quality of the trimmed filtered reads was revaluated using FastQC (v0.11.9). The trimmed reads were then aligned to the human reference genome GRCh38, of which more than 95% of the paired reads were successfully mapped using the Burrows–Wheeler Aligner (BWA) software (v0.7.17) in the paired-end mode of the BWA-MEM algorithm [94] with the default parameter settings. The mapped read files (*.sam) were subsequently converted to sorted compressed binary version BAM files by SAMtools (v1.11) [95]. Only those reads that mapped to the reference genome were considered for further analysis, the unmapped reads were filtered out using SAMtools (v1.11) [95].

We then used the Bioconductor package MEDIPS (v1.40.0) [96] on our mapped reads to perform quality control tests on the MeDIP-seq data. A Pearson’s correlation test was performed on the mapped reads among the biological replicates to ensure the reproducibility of the results. High correlation scores of > 0.99 were obtained across the four biological replicates within each irradiation dose across the SF and MF exposures. Saturation analysis was further conducted on the data to determine whether a sufficient number of reads was generated for a genome-wide coverage to yield reliable and reproducible methylome profiles of each DNA sample. The saturation analysis was carried out using three different window sizes: 50 bp, 100 bp, and 150 bp. The average score for the 3 windows across all samples was > 99%. A window size of 50 bp was later used in the downstream analysis for high-resolution methylome profiles while still maintaining high sequencing depth. On average, > 91% of total CpG dinucleotides were covered in our study.

The MEDIPS (v1.40.0) [96] analysis package was also used to identify DMRs between controls and irradiated cells for both regimens. First, datasets were created using a genome window size of 50 bp (ws = 50) without any shifting of the reads in the genomic locations (shift = 0). The extend parameter was neglected since the actual DNA fragment length is known in paired-end sequenced data. Only one representative read mapped to the same genomic position was considered in this study by setting the parameter “uniq” to 1 to avoid false positive differential enrichment between conditions. Quality control specific to immunoprecipitated enriched data, including saturation analysis, CpG coverage analysis and enrichment analysis, was conducted in all samples across both conditions in the α-irradiated samples. Differential coverage was calculated using the edgeR function in MEDIPS with a minimum of 5 reads per 50 bp window across replicates (minRowSum = 5). Normalization of both library sizes and enrichment efficiencies was carried out using the quantile parameter. P values were adjusted for multiple testing using the False Discovery Rate (FDR) method. Statistically significant DNAm coverage results were selected using the criteria of FDR < 0.05. Additional file 1: Table S3 displays the number of DMRs calculated in the irradiated cells compared with the controls using different filters for statistical significance. Minus average plots were generated to illustrate the number of DMRs identified in each of the irradiated groups using different statistical significance criteria (Additional file 1: Figure S1). The resulting DMRs underwent annotation based on their corresponding genomic regions using HOMER (v4.11) annotatePeaks [97]. Neighboring significant regions located within the same chromosome, and with the same annotation and Gene ID, were merged using an in-house Python script. Merged DMRs were then used for gene ontology and pathway enrichment analysis using the PANTHER algorithm (v16.0) [98–100] and IPA software. We also examined the distribution of the DMRs across all chromosomes including both nuclear and mitochondrial DNA identified in the α-irradiated fibroblasts. Feature sets were subsequently defined spanning sub-typed by genomic location, including exon, intron, promoter-transcription start site (referred to as promoter), 3’ untranslated region, 5’ untranslated region, transcription termination site, and DNA repeats, of the DMRs in the α-irradiated groups.

### Supplementary Information

**Additional file 1.** **Figure S1:** Minus-Average plots illustrating the number of differentially methylated regions (DMRs) identified using various filters for statistical significance in α-irradiated fibroblasts. A DMRs detected in fibroblasts irradiated to single-fraction doses of α-particles. B DMRs detected in fibroblasts irradiated to multi-fraction doses of α-particles. Red, green, and gold dots indicate enriched regions with adjusted p value < 0.05, adjusted p value < 0.1, and raw p value <0.05, respectively. P values were adjusted for multiple testing using the false discovery rate method. **Table S1:** Parameters of the single-fraction α-irradiation using americium-241 sources in lung fibroblast cells. **Table S2:** Parameters of the 14-day multi-fraction α-irradiation equally delivered every 24 hours using americium-241 sources in lung fibroblast cells. **Table S3:** Number of differentially methylated regions (DMRs) in α-irradiated fibroblasts. The dose was delivered either as a singlefraction or equally distributed in 14 fractions (multi-fraction) with one fraction per day every 24 hours. The DMRs were generated using the MEDIPS package. The adjusted p values were computed using the false discovery rate (FDR) method. **Table S4:** Genes harboring the aging-associated differentially methylated regions (DMRs) in the α-irradiated lung fibroblasts. The total dose was delivered either as a single-fraction (SF) or 14-d multi-fraction (MF) every 24 hours. These events are based on the epigenetic chronological DNAm clock from Horvath [33] and the biological DNAm clock from Levine, et al. [34]. HypoDMRs, hypomethylated DMRs; hyperDMRs, hypermethylated DMRs; chr, chromosome. **Table S5:** The list of all differentially methylated regions (DMRs) located within the promoter site of the genome and their associated genes identified in the lung fibroblasts irradiated to single-fraction (SF) doses of α-particles. HypoDMRs, hypomethylated DMRs; HyperDMRs, hypermethylated DMRs; chr, chromosome. **Table S6:** The list of all differentially methylated regions (DMRs) located within the promoter site of the genome and their associated genes identified in the lung fibroblasts irradiated to multi-fraction (MF) doses of α-particles. Each fraction was equally delivered every 24 hours for 14 days. HypoDMRs, hypomethylated DMRs; HyperDMRs, hypermethylated DMRs; chr, chromosome.

## Data Availability

The MeDIP-seq data from primary lung fibroblasts from control and irradiated to a wide range of doses of α-particles reported in this paper are deposited in Gene Expression Omnibus (GEO) database under accession number GSE242767 (https://www.ncbi.nlm.nih.gov/geo/query/acc.cgi?acc=GSE242767).

## Abbreviations

α: Alpha
DNAm: DNA methylation
SF: Single-fraction
MF: Multi-fraction
^222^Rn: Radon-222
CpG: Cytosine and guanine
5mC: 5-Methylcytosine
SF^2.0^: 2.0 mGy α-irradiation in a SF
SF^11^: 11 mGy α-irradiation in a SF
SF^23^: 23 mGy α-irradiation in a SF
SF^110^: 110 mGy α-irradiation in a SF
SF^220^: 220 mGy α-irradiation in a SF
SF^1100^: 1,100 mGy α-irradiation in a SF
SF^2200^: 2,200 mGy α-irradiation in a SF
MF^2.0^: 2.0 mGy α-irradiation delivered in MF
MF^11^: 11 mGy α-irradiation delivered in MF
MF^23^: 23 mGy α-irradiation delivered in MF
MF^110^: 110 mGy α-irradiation delivered in MF
MF^220^: 220 mGy α-irradiation delivered in MF
MF^1100^: 1,100 mGy α-irradiation delivered in MF
MF^2200^: 2,200 mGy α-irradiation delivered in MF
MeDIP-seq: Methylated DNA immunoprecipitation coupled with deep sequencing
DMRs: Differentially methylated regions
hypoDMRs: Hypomethylated DMRs
hyperDMRs: Hypermethylated DMRs
IPA: Ingenuity Pathway Analysis
bp: Basepair
*NSDHL*: NAD(P)-dependent steroid dehydrogenase-like
*SLC16A3*: Solute carrier family 16 member 3
*CPNE1*: Copine 1
*LIFR*: LIF receptor subunit alpha
*PRKACG*: Protein kinase CAMP-activated catalytic subunit gamma
*CAMK2A*: Calcium/calmodulin-dependent protein kinase II alpha
*LINC00884*: Long intergenic non-protein coding RNA 884
*LINC01624*: Long intergenic non-protein coding RNA 1624
*MAGEA12*: Melanoma-associated antigen A12
*PRPS2*: Phosphoribosyl pyrophosphate synthetase 2
*ZDHHC16*: Zinc finger DHHC-type palmitoyltransferase 16
*RHOT2*: *ras* Homolog family member T2
*LINC02280*: Long intergenic non-protein coding RNA 2280
*FES*: FES proto-oncogene
*ACRBP*: Acrosin binding protein
*GALNT9*: UDP-GalNAc: polypeptide N-acetylgalactosaminyltransferase 9
*MPP2*: MAGUK p55 scaffold protein 2
*EVI5L*: Ecotropic viral integration site 5-like
*ATP8B3*: ATPase phospholipid transporting 8b3
*ABCA7*: ATP-binding cassette subfamily A member 7
*BCAM*: Basal cell adhesion molecule
*ARHGEF16*: Rho guanine nucleotide exchange factor 16
*PLCH2*: Phospholipase C eta 2
*JAK2*: Janus kinase 2
*MGMT*: O^6^-methylguanine-DNA methyltransferase
*RB1*: RB transcriptional corepressor 1
*CRYAB*: Crystallin alpha B
*F2R*: Coagulation factor II thrombin receptor
*PTGDS*: Prostaglandin D2 synthase
*SCN4A*: Sodium voltage-gated channel alpha subunit 4
*FANCG*: FA complementation group G
*GPX4*: Glutathione peroxidase 4
LET: Linear energy transfer
FDR: False discovery rate
