# Supplementary material for: Profound DNA methylomic differences between single- and multi-fraction alpha irradiations of lung fibroblasts
Source: Clin Epigenetics. 2023 Oct 27;15:174. doi: 10.1186/s13148-023-01564-z (PMC10612361; doi:10.1186/s13148-023-01564-z)
Supplement: Supplementary file 1 — Additional file 1. Figure S1: Minus-Average plots illustrating the number of differentially methylated regions (DMRs) identified using various filters for statistical significance in α-irradiated fibroblasts. A DMRs detected in fibroblasts irradiated to single-fraction doses of α-particles. B DMRs detected in fibroblasts irradiated to multi-fraction doses of α-particles. Red, green, and gold dots indicate enriched regions with adjusted p value < 0.05, adjusted p value < 0.1, and raw p value <0.05, respectively. P values were adjusted for multiple testing using the false discovery rate method. Table S1: Parameters of the single-fraction α-irradiation using americium-241 sources in lung fibroblast cells. Table S2: Parameters of the 14-day multi-fraction α-irradiation equally delivered every 24 hours using americium-241 sources in lung fibroblast cells. Table S3: Number of differentially methylated regions (DMRs) in α-irradiated fibroblasts. The dose was delivered either as a singlefraction or equally distributed in 14 fractions (multi-fraction) with one fraction per day every 24 hours. The DMRs were generated using the MEDIPS package. The adjusted p values were computed using the false discovery rate (FDR) method. Table S4: Genes harboring the aging-associated differentially methylated regions (DMRs) in the α-irradiated lung fibroblasts. The total dose was delivered either as a single-fraction (SF) or 14-d multi-fraction (MF) every 24 hours. These events are based on the epigenetic chronological DNAm clock from Horvath [33] and the biological DNAm clock from Levine, et al. [34]. HypoDMRs, hypomethylated DMRs; hyperDMRs, hypermethylated DMRs; chr, chromosome. Table S5: The list of all differentially methylated regions (DMRs) located within the promoter site of the genome and their associated genes identified in the lung fibroblasts irradiated to single-fraction (SF) doses of α-particles. HypoDMRs, hypomethylated DMRs; HyperDMRs, hypermethylated DMRs; chr, chromosome. T [file 13148_2023_1564_MOESM1_ESM.docx]

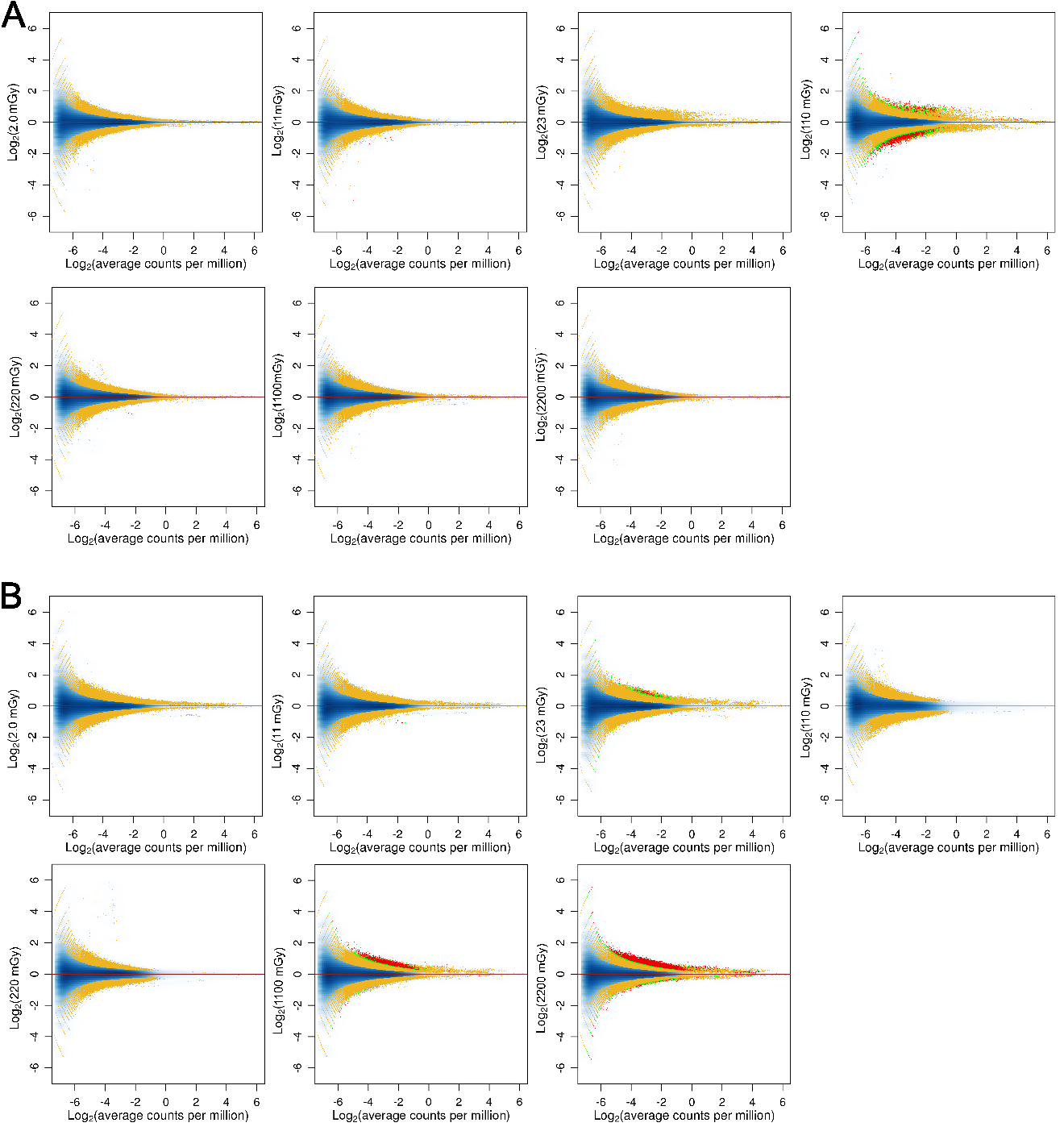


Figure S1. Minus-Average plots illustrating the number of differentially methylated regions (DMRs) identified using various filters for statistical significance in α-irradiated fibroblasts. A DMRs detected in fibroblasts irradiated to single-fraction doses of α-particles. B DMRs detected in fibroblasts irradiated to multi-fraction doses of α-particles. Red, green, and gold dots indicate enriched regions with adjusted p value < 0.05, adjusted p value < 0.1, and raw p value <0.05, respectively. P values were adjusted for multiple testing using the false discovery rate method.

Table S1. Parameters of the single-fraction α-irradiation using americium-241 sources in lung fibroblast cells.

| **Dose Designation^1^ (mGy)** | **Dose Rate (mGy·s^-1^)** | **Irradiation Time^2^ (s)** | **Dose Delivered^3^ (mGy)** |
| --- | --- | --- | --- |
| 2.0 | 0.14 ± 0.005 | 14 | 1.908 ± 0.065 |
| 11 | 0.14 ± 0.005 | 84 | 11.45 ± 0.39 |
| 23 | 0.14 ± 0.005 | 168 | 22.89 ± 0.77 |
| 110 | 2.6 ± 0.09 | 42 | 109.3 ± 3.9 |
| 220 | 2.6 ± 0.09 | 84 | 218.7 ± 7.9 |
| 1100 | 2.6 ± 0.09 | 420 | 1093 ± 39 |
| 2200 | 2.6 ± 0.09 | 840 | 2187 ± 79 |
| ^1^Doses as referred to in the text. They have been rounded to 2 significant figures.  ^2^The amount of time the americium-241 sources were kept over the sample surface to achieve the intended dose.  ^3^The total single-fraction dose delivered during the irradiation day. | | | |

Table S2. Parameters of the 14-day multi-fraction α-irradiation equally delivered every 24 hours using americium-241 sources in lung fibroblast cells.

| **Dose Designation^1^ (mGy)** | **Dose Rate (mGy·s^−1^)** | **Irradiation Time^2^ (s)** | **Dose per Fraction^3^ (mGy·d^−1^)** | **Total Dose Delivered^4^ (mGy)** |
| --- | --- | --- | --- | --- |
| 2.0 | 0.14 ± 0.005 | 1 | 0.136 ± 0.005 | 1.908 ± 0.065 |
| 11 | 0.14 ± 0.005 | 6 | 0.818 ± 0.03 | 11.45 ± 0.39 |
| 23 | 0.14 ± 0.005 | 12 | 1.64 ± 0.06 | 22.89 ± 0.77 |
| 110 | 2.6 ± 0.09 | 3 | 7.81 ± 0.3 | 109.3 ± 3.9 |
| 220 | 2.6 ± 0.09 | 6 | 15.6 ± 0.6 | 218.7 ± 7.9 |
| 1100 | 2.6 ± 0.09 | 30 | 78.1 ± 3 | 1093 ± 39 |
| 2200 | 2.6 ± 0.09 | 60 | 156 ± 6 | 2187 ± 79 |
| ^1^Doses as referred to in the text. They have been rounded to 2 significant figures.  ^2^The amount of time the americium-241 sources were daily kept over the sample surface.  ^3^The actual dose per fraction administered daily to the samples.  ^4^The total dose delivered following a 14-day consecutive irradiation every 24 hours. | | | | |

Table S3. Number of differentially methylated regions (DMRs) in α-irradiated fibroblasts. The dose was delivered either as a single-fraction or equally distributed in 14 fractions (multi-fraction) with one fraction per day every 24 hours. The DMRs were generated using the MEDIPS package. The adjusted p values were computed using the false discovery rate (FDR) method.

| **Total dose**  **(mGy)** | **Single-fraction Exposure** | | | | **Multi-fraction Exposure** | | | |
| --- | --- | --- | --- | --- | --- | --- | --- | --- |
|  | **Raw**  **p value**  **<0.05** | **Adjusted**  **p value**  **<0.1** | **Adjusted**  **p value**  **<0.05** | **Merged**  **FDR**  **<0.05** | **Raw**  **p value**  **<0.05** | **Adjusted**  **p value**  **<0.1** | **Adjusted**  **p value**  **<0.05** | **Merged**  **FDR**  **<0.05** |
| 2.0 | 634,582 | 1 | 0 | 0 | 726,970 | 1 | 1 | 1 |
| 11 | 581,195 | 13 | 10 | 6 | 661,216 | 9 | 5 | 4 |
| 23 | 721,630 | 0 | 0 | 0 | 853,693 | 401 | 102 | 83 |
| 110 | 877,339 | 3,224 | 1,250 | 920 | 549,944 | 0 | 0 | 0 |
| 220 | 598,368 | 3 | 3 | 1 | 441,678 | 0 | 0 | 0 |
| 1,100 | 596,845 | 0 | 0 | 0 | 758,915 | 3,016 | 1,342 | 1,044 |
| 2,200 | 524,270 | 0 | 0 | 0 | 853,309 | 12,579 | 6,662 | 4,039 |

Table S4. Genes harboring the aging-associated differentially methylated regions (DMRs) in the α-irradiated lung fibroblasts. The total dose was delivered either as a single-fraction (SF) or 14-d multi-fraction (MF) every 24 hours. These events are based on the epigenetic chronological DNAm clock from Horvath [33] and the biological DNAm clock from Levine, et al. [34]. HypoDMRs, hypomethylated DMRs; hyperDMRs, hypermethylated DMRs; chr, chromosome.

| **Group** | **Chr** | **Entrez ID** | **Gene Symbol** | **Annotation** | **Horvath [33]** | **Levine, et al. [34]** |
| --- | --- | --- | --- | --- | --- | --- |
| HypoDMRs SF^110^ | chr1 | 339479 | BRINP3 | Intergenic |  | Yes |
|  | chr1 | 3061 | HCRTR1 | exon | Yes |  |
|  | chr3 | 10015 | PDCD6IP | Intergenic | Yes |  |
|  | chr5 | 83734 | ATG10 | Intron |  | Yes |
|  | chr5 | 1007 | CDH9 | Intergenic |  | Yes |
|  | chr6 | 7957 | EPM2A | Intergenic | Yes |  |
|  | chr7 | 63974 | NEUROD6 | Intergenic |  | Yes |
|  | chr7 | 55695 | NSUN5 | Intergenic | Yes |  |
|  | chr8 | 54845 | ESRP1 | intron |  | Yes |
|  | chr9 | 153090 | DAB2IP | intron | Yes |  |
|  | chr10 | 4255 | MGMT | intron |  | Yes |
|  | chr14 | 2972 | BRF1 | intron |  | Yes |
|  | chr15 | 23191 | CYFIP1 | intron | Yes |  |
|  | chr16 | 5347 | PLK1 | exon/intron | Yes |  |
|  | chr17 | 40 | ASIC2 | intron | Yes |  |
|  | chr17 | 10594 | PRPF8 | intron | Yes |  |
|  | chr19 | 23370 | ARHGEF18 | exon | Yes |  |
|  | chr19 | 3643 | INSR | exon |  | Yes |
|  | chr21 | 150094 | SIK1 | intron |  | Yes |
| HyperDMRs SF^110^ | chr3 | 27094 | KCNMB3 | intron |  | Yes |
|  |  |  |  |  |  |  |
| HypoDMRs MF^2200^ | chr5 | 902 | CCNH | Intergenic | Yes |  |
|  | chr8 | 51312 | SLC25A37 | intron |  | Yes |
|  | chr11 | 6888 | TALDO1 | intron |  | Yes |
| HyperDMRs MF^23^ | chr6 | 860 | RUNX2 | intron |  | Yes |
|  | chr9 | 80709 | AKNA | intron |  | Yes |
|  | chr11 | 57053 | CHRNA10 | exon |  | Yes |
|  | chr15 | 2242 | FES | exon/promoter-TSS | Yes |  |
|  | chr19 | 4059 | BCAM | exon | Yes |  |
| HyperDMRs MF^1100^ | chr1 | 8659 | ALDH4A1 | intron | Yes |  |
|  | chr1 | 284723 | SLC25A34 | exon/intron |  | Yes |
|  | chr1 | 8718 | TNFRSF25 | intron |  | Yes |
|  | chr2 | 2736 | GLI2 | intron | Yes |  |
|  | chr2 | 23620 | NTSR2 | Intergenic | Yes |  |
|  | chr4 | 2868 | GRK4 | intron |  | Yes |
|  | chr4 | 7466 | WFS1 | intron | Yes |  |
|  | chr5 | 815 | CAMK2A | promoter-TSS |  | Yes |
|  | chr9 | 153090 | DAB2IP | exon | Yes |  |
|  | chr9 | 3933 | LCN1 | intron | Yes |  |
|  | chr9 | 9933 | PUM3 | Intergenic | Yes |  |
|  | chr9 | 6256 | RXRA | Intergenic/intron | Yes |  |
|  | chr9 | 3371 | TNC | exon | Yes |  |
|  | chr11 | 11041 | B4GAT1 | exon |  | Yes |
|  | chr11 | 57053 | CHRNA10 | exon |  | Yes |
|  | chr11 | 5936 | RBM4 | exon |  | Yes |
|  | chr11 | 6484 | ST3GAL4 | exon/intron | Yes |  |
|  | chr12 | 4326 | MMP17 | TTS |  | Yes |
|  | chr12 | 9271 | PIWIL1 | intron |  | Yes |
|  | chr13 | 23263 | MCF2L | intron |  | Yes |
|  | chr14 | 2972 | BRF1 | intron |  | Yes |
|  | chr14 | 23186 | RCOR1 | intron |  | Yes |
|  | chr15 | 290 | ANPEP | intron |  | Yes |
|  | chr15 | 2242 | FES | exon/promoter-TSS | Yes |  |
|  | chr15 | 7082 | TJP1 | intron | Yes |  |
|  | chr16 | 80270 | HSD3B7 | TTS |  | Yes |
|  | chr16 | 9100 | USP10 | intron | Yes | Yes |
|  | chr17 | 60528 | ELAC2 | intron | Yes |  |
|  | chr17 | 5606 | MAP2K3 | Intergenic |  | Yes |
|  | chr17 | 4641 | MYO1C | intron |  | Yes |
|  | chr17 | 10594 | PRPF8 | Intergenic | Yes |  |
|  | chr18 | 3909 | LAMA3 | intron | Yes |  |
|  | chr19 | 339366 | ADAMTSL5 | exon |  | Yes |
|  | chr19 | 4059 | BCAM | exon/intron | Yes |  |
|  | chr19 | 682 | BSG | intron |  | Yes |
|  | chr19 | 148113 | CILP2 | Intergenic |  | Yes |
|  | chr19 | 51343 | FZR1 | intron | Yes |  |
|  | chr19 | 29985 | SLC39A3 | TTS |  | Yes |
|  | chr19 | 8677 | STX10 | exon | Yes |  |
|  | chr19 | 55850 | USE1 | exon |  | Yes |
|  | chr20 | 22981 | NINL | intron | Yes |  |
|  | chr20 | 140688 | NOL4L | intron |  | Yes |
|  | chr21 | 8208 | CHAF1B | intron | Yes | Yes |
|  | chr21 | 150094 | SIK1 | Intergenic |  | Yes |
| HyperDMRs MF^2200^ | chr1 | 81569 | ACTL8 | exon/intron |  | Yes |
|  | chr1 | 8659 | ALDH4A1 | exon | Yes |  |
|  | chr1 | 339479 | BRINP3 | intergenic |  | Yes |
|  | chr1 | 1063 | CENPF | intron | Yes |  |
|  | chr1 | 9077 | DIRAS3 | promoter-TSS | Yes |  |
|  | chr1 | 128178 | EDARADD | intron | Yes | Yes |
|  | chr1 | 26270 | FBXO6 | exon |  | Yes |
|  | chr1 | 3339 | HSPG2 | exon/intron | Yes |  |
|  | chr1 | 79605 | PGBD5 | 3' UTR |  | Yes |
|  | chr1 | 57449 | PLEKHG5 | intron |  | Yes |
|  | chr1 | 284723 | SLC25A34 | exon/intron |  | Yes |
|  | chr1 | 339488 | TFAP2E | exon | Yes |  |
|  | chr1 | 8718 | TNFRSF25 | intron |  | Yes |
|  | chr1 | 29089 | UBE2T | exon |  | Yes |
|  | chr2 | 250 | ALPP | intergenic | Yes |  |
|  | chr2 | 9759 | HDAC4 | intron |  | Yes |
|  | chr2 | 3973 | LHCGR | TTS | Yes |  |
|  | chr2 | 23620 | NTSR2 | exon/intron | Yes |  |
|  | chr2 | 84236 | RHBDD1 | 3' UTR | Yes | Yes |
|  | chr3 | 9435 | CHST2 | intergenic | Yes |  |
|  | chr3 | 1181 | CLCN2 | intron | Yes |  |
|  | chr3 | 2912 | GRM2 | exon | Yes |  |
|  | chr3 | 27094 | KCNMB3 | intergenic |  | Yes |
|  | chr3 | 54800 | KLHL24 | intergenic |  | Yes |
|  | chr3 | 51460 | SFMBT1 | intron | Yes | Yes |
|  | chr3 | 7047 | TGM4 | exon |  | Yes |
|  | chr3 | 54106 | TLR9 | 3' UTR |  | Yes |
|  | chr4 | 27065 | NSG1 | intron |  | Yes |
|  | chr4 | 7466 | WFS1 | intron | Yes |  |
|  | chr5 | 815 | CAMK2A | promoter-TSS |  | Yes |
|  | chr5 | 1007 | CDH9 | intergenic |  | Yes |
|  | chr5 | 8817 | FGF18 | 3' UTR/intron | Yes |  |
|  | chr5 | 51237 | MZB1 | 3' UTR | Yes |  |
|  | chr5 | 80758 | PRR7 | TTS |  | Yes |
|  | chr5 | 79770 | TXNDC15 | intron | Yes | Yes |
|  | chr6 | 51250 | C6orf203 | exon |  | Yes |
|  | chr6 | 667 | DST | intron | Yes |  |
|  | chr6 | 80069 | LINC00574 | exon | Yes |  |
|  | chr7 | 8326 | FZD9 | exon | Yes |  |
|  | chr7 | 6608 | SMO | exon |  | Yes |
|  | chr8 | 444 | ASPH | intron |  | Yes |
|  | chr8 | 55806 | HR | exon | Yes |  |
|  | chr8 | 6482 | ST3GAL1 | intron |  | Yes |
|  | chr8 | 23087 | TRIM35 | exon |  | Yes |
|  | chr9 | 11094 | CACFD1 | exon |  | Yes |
|  | chr9 | 153090 | DAB2IP | exon/intron | Yes |  |
|  | chr9 | 57171 | DOLPP1 | exon/intron | Yes |  |
|  | chr9 | 3933 | LCN1 | exon/intron | Yes |  |
|  | chr9 | 56654 | NPDC1 | intron |  | Yes |
|  | chr9 | 9933 | PUM3 | intergenic | Yes |  |
|  | chr9 | 6256 | RXRA | intergenic/intron | Yes |  |
|  | chr9 | 3371 | TNC | exon | Yes |  |
|  | chr9 | 7539 | ZFP37 | intergenic |  | Yes |
|  | chr10 | 240 | ALOX5 | intron |  | Yes |
|  | chr10 | 5309 | PITX3 | intron | Yes |  |
|  | chr10 | 219793 | TBATA | exon/intron | Yes |  |
|  | chr10 | 118472 | ZNF511 | intron |  | Yes |
|  | chr11 | 221 | ALDH3B1 | intron | Yes |  |
|  | chr11 | 11041 | B4GAT1 | exon |  | Yes |
|  | chr11 | 8722 | CTSF | exon |  | Yes |
|  | chr11 | 8642 | DCHS1 | exon | Yes |  |
|  | chr11 | 4054 | LTBP3 | exon | Yes |  |
|  | chr11 | 4607 | MYBPC3 | exon | Yes |  |
|  | chr11 | 5936 | RBM4 | exon |  | Yes |
|  | chr11 | 6484 | ST3GAL4 | exon | Yes |  |
|  | chr12 | 4326 | MMP17 | exon/TTS |  | Yes |
|  | chr12 | 9271 | PIWIL1 | exon |  | Yes |
|  | chr12 | 59341 | TRPV4 | exon/promoter-TSS |  | Yes |
|  | chr13 | 23263 | MCF2L | exon/intron |  | Yes |
|  | chr14 | 2972 | BRF1 | exon/intron |  | Yes |
|  | chr14 | 8111 | GPR68 | exon | Yes |  |
|  | chr14 | 64423 | INF2 | intron |  | Yes |
|  | chr14 | 23186 | RCOR1 | intron |  | Yes |
|  | chr15 | 23191 | CYFIP1 | exon/intron | Yes |  |
|  | chr15 | 2242 | FES | exon/intron | Yes |  |
|  | chr15 | 54551 | MAGEL2 | exon |  | Yes |
|  | chr15 | 7026 | NR2F2 | intergenic | Yes |  |
|  | chr16 | 21 | ABCA3 | exon | Yes |  |
|  | chr16 | 3029 | HAGH | intron |  | Yes |
|  | chr16 | 80270 | HSD3B7 | TTS |  | Yes |
|  | chr16 | 57338 | JPH3 | intergenic | Yes |  |
|  | chr16 | 197257 | LDHD | exon/intron |  | Yes |
|  | chr16 | 161882 | ZFPM1 | intergenic |  | Yes |
|  | chr17 | 84254 | CAMKK1 | exon/intron |  | Yes |
|  | chr17 | 57513 | CASKIN2 | 5' UTR | Yes |  |
|  | chr17 | 5606 | MAP2K3 | Intergenic/3' UTR |  | Yes |
|  | chr17 | 4641 | MYO1C | intron |  | Yes |
|  | chr17 | 10400 | PEMT | intron |  | Yes |
|  | chr17 | 10594 | PRPF8 | intron | Yes |  |
|  | chr18 | 2587 | GALR1 | intergenic | Yes |  |
|  | chr18 | 80148 | SLC66A2 | exon/intron | Yes |  |
|  | chr19 | 115703 | ARHGAP33 | intron |  | Yes |
|  | chr19 | 23370 | ARHGEF18 | exon/intron | Yes |  |
|  | chr19 | 4059 | BCAM | exon/intron | Yes |  |
|  | chr19 | 148113 | CILP2 | intron |  | Yes |
|  | chr19 | 51343 | FZR1 | intron | Yes |  |
|  | chr19 | 10365 | KLF2 | intron | Yes | Yes |
|  | chr19 | 3972 | LHB | intron |  | Yes |
|  | chr19 | 4713 | NDUFB7 | intron/TTS | Yes |  |
|  | chr19 | 4784 | NFIX | intron |  | Yes |
|  | chr19 | 5143 | PDE4C | exon | Yes | Yes |
|  | chr19 | 5817 | PVR | exon |  | Yes |
|  | chr19 | 29985 | SLC39A3 | TTS |  | Yes |
|  | chr19 | 8677 | STX10 | exon/intron | Yes |  |
|  | chr19 | 7089 | TLE2 | 3' UTR | Yes |  |
|  | chr19 | 9322 | TRIP10 | TTS | Yes |  |
|  | chr19 | 55850 | USE1 | exon/intron |  | Yes |
|  | chr20 | 140836 | BANF2 | intergenic |  | Yes |
|  | chr20 | 22981 | NINL | intron | Yes |  |
|  | chr20 | 11317 | RBPJL | exon/intron |  | Yes |
|  | chr20 | 54453 | RIN2 | intron | Yes | Yes |
|  | chr21 | 150094 | SIK1 | exon/intergenic/intron |  | Yes |
|  | chr22 | 1454 | CSNK1E | exon |  | Yes |
|  | chr22 | 4330 | MN1 | intergenic | Yes |  |
|  | chr18 | 80148 | SLC66A2 | exon/intron | Yes |  |

Table S5. The list of all differentially methylated regions (DMRs) located within the promoter site of the genome and their associated genes identified in the lung fibroblasts irradiated to single-fraction (SF) doses of α-particles. HypoDMRs, hypomethylated DMRs; HyperDMRs, hypermethylated DMRs; chr, chromosome.

| Group | Chr | Entrez ID | Gene Symbol | Gene name |
| --- | --- | --- | --- | --- |
| HypoDMRs SF^11^ | Chr21 | 100500862 | MIR3648-1 | microRNA 3648-1 |
|  | Chr22 | 109864282 | RNA28SN2 | RNA, 5.8S ribosomal N1 |
|  | Chr22 | 106632260 | RNA5-8SN1 | RNA, 28S ribosomal N2 |
| HypoDMRs SF^110^ | chr5 | 3977 | LIFR | LIF receptor subunit alpha |
|  | chr5 | 729862 | LSP1P3 | LSP1 pseudogene 3 |
|  | chr7 | 102465504 | MIR6838 | microRNA 6838 |
|  | chr8 | 286076 | BREA2 | breast cancer estrogen-induced apoptosis 2 |
|  | chr9 | 5568 | PRKACG | protein kinase cAMP-activated catalytic subunit gamma |
|  | chr9 | 2189 | FANCG | FA complementation group G |
|  | chr11 | 55231 | CCDC87 | coiled-coil domain containing 87 |
|  | chr11 | 102465451 | MIR6753 | microRNA 6753 |
|  | chr13 | 102723345 | LOC102723345 | uncharacterized LOC102723345 |
|  | chr16 | 100128882 | LOC100128882 | uncharacterized LOC100128882 |
|  | chr16 | 645644 | FLJ42627 | uncharacterized LOC645644 |
|  | chr17 | 79755 | ZNF750 | zinc finger protein 750 |
|  | chr17 | 9123 | SLC16A3 | solute carrier family 16 member 3 |
|  | chr19 | 100170227 | SNAR-D | small NF90 (ILF3) associated RNA D |
|  | chr20 | 26013 | L3MBTL1 | L3MBTL histone methyl-lysine binding protein 1 |
|  | chr20 | 8904 | CPNE1 | copine 1 |
|  | chrX | 50814 | NSDHL | NAD(P) dependent steroid dehydrogenase-like |
| HypoDMRs SF^220^ | chr22 | 106632260 | RNA5-8SN1 | RNA, 5.8S ribosomal N1 |
| HyperDMRs SF^110^ | chr6 | 4201 | MEA1 | male-enhanced antigen 1 |
|  | chr11 | 975 | CD81 | CD81 molecule |
|  | chr14 | 51527 | GSKIP | GSK3B interacting protein |
|  | chr18 | 727758 | ROCK1P1 | Rho associated coiled-coil containing protein kinase 1 pseudogene 1 |
|  | chr21 | 103504733 | MIR6724-4 | microRNA 6724-4 |
|  | chr21 | 102465433 | MIR6724-1 | microRNA 6724-1 |
|  | chr22 | 109864282 | RNA28SN2 | RNA, 28S ribosomal N2 |
|  | chr22 | 109864271 | RNA45SN4 | RNA, 45S pre-ribosomal N4 |
|  | chrUn | 109864274 | RNA5-8SN4 | RNA, 5.8S ribosomal N4 |

Table S6. The list of all differentially methylated regions (DMRs) located within the promoter site of the genome and their associated genes identified in the lung fibroblasts irradiated to multi-fraction (MF) doses of α-particles. Each fraction was equally delivered every 24 hours for 14 days. HypoDMRs, hypomethylated DMRs; HyperDMRs, hypermethylated DMRs; chr, chromosome.

| **Group** | **Chr** | **Entrez ID** | **Gene Symbol** | **Gene name** |
| --- | --- | --- | --- | --- |
| HypoDMRs MF^11^ | chr22 | 106632260 | RNA5-8SN1 | RNA, 5.8S ribosomal N1 |
| HypoDMRs MF^2200^ | chr17 | 90507 | SCRN2 | secernin 2 |
| HyperDMRs MF^23^ | chr9 | 26086 | GPSM1 | G protein signaling modulator 1 |
|  | chr14 | 101929634 | LINC02280 | long intergenic non-protein coding RNA 2280 |
|  | chr15 | 2242 | FES | FES proto-oncogene, tyrosine kinase |
|  | chr16 | 89941 | RHOT2 | ras homolog family member T2 |
| HyperDMRs MF^1100^ | chr1 | 54936 | ADPRHL2 | ADP-ribosylhydrolase like 2 |
|  | chr1 | 676 | BRDT | bromodomain testis associated |
|  | chr1 | 4802 | NFYC | nuclear transcription factor Y subunit gamma |
|  | chr3 | 401106 | LINC00884 | long intergenic non-protein coding RNA 884 |
|  | chr5 | 815 | CAMK2A | calcium/calmodulin dependent protein kinase II alpha |
|  | chr5 | 206338 | LVRN | laeverin |
|  | chr6 | 401289 | LINC01624 | long intergenic non-protein coding RNA 1624 |
|  | chr7 | 109623488 | SNORA114 | small nucleolar RNA, H/ACA box 114 |
|  | chr8 | 5339 | PLEC | plectin |
|  | chr8 | 619554 | MIR486-1 | microRNA 486-1 |
|  | chr9 | 29952 | DPP7 | dipeptidyl peptidase 7 |
|  | chr10 | 84287 | ZDHHC16 | zinc finger DHHC-type containing 16 |
|  | chr10 | 9721 | GPRIN2 | G protein regulated inducer of neurite outgrowth 2 |
|  | chr10 | 10844 | TUBGCP2 | tubulin gamma complex associated protein 2 |
|  | chr11 | 283120 | H19 | H19 imprinted maternally expressed transcript |
|  | chr12 | 57696 | DDX55 | DEAD-box helicase 55 |
|  | chr13 | 220429 | CTAGE10P | CTAGE family member 10, pseudogene |
|  | chr13 | 3916 | LAMP1 | lysosomal associated membrane protein 1 |
|  | chr15 | 2242 | FES | FES proto-oncogene, tyrosine kinase |
|  | chr16 | 348174 | CLEC18A | C-type lectin domain family 18 member A |
|  | chr16 | 104644205 | ENPP7P13 | ectonucleotide pyrophosphatase/phosphodiesterase 7 pseudogene 13 |
|  | chr17 | 284123 | FAM27E5 | family with sequence similarity E5 |
|  | chr17 | 10801 | SEPTIN9 | septin 9 |
|  | chr17 | 102465470 | MIR6784 | microRNA 6784 |
|  | chr18 | 64693 | CTAGE1 | cutaneous T cell lymphoma-associated antigen 1 |
|  | chr20 | 55861 | DBNDD2 | dysbindin domain containing 2 |
|  | chr22 | 7152 | TOP1P2 | DNA topoisomerase I pseudogene 2 |
|  | chrX | 4111 | MAGEA12 | MAGE family member A12 |
|  | chrX | 5634 | PRPS2 | phosphoribosyl pyrophosphate synthetase 2 |
|  | chrX | 83550 | GPR101 | G protein-coupled receptor 101 |
|  | chrX | 8517 | IKBKG | inhibitor of nuclear factor kappa B kinase regulatory subunit gamma |
| HyperDMRs MF^2200^ | chr1 | 102465434 | MIR6726 | microRNA 6726 |
|  | chr1 | 693135 | MIR551A | microRNA 551a |
|  | chr1 | 100132147 | LINC01783 | long intergenic non-protein coding RNA 1783 |
|  | chr1 | 9651 | PLCH2 | phospholipase C eta 2 |
|  | chr1 | 4802 | NFYC | nuclear transcription factor Y subunit gamma |
|  | chr1 | 9077 | DIRAS3 | DIRAS family GTPase 3 |
|  | chr1 | 731656 | LINC01348 | long intergenic non-protein coding RNA 1348 |
|  | chr2 | 200772 | LOC200772 | uncharacterized LOC200772 |
|  | chr2 | 10267 | RAMP1 | receptor activity modifying protein 1 |
|  | chr2 | 23160 | WDR43 | WD repeat domain 43 |
|  | chr2 | 101805491 | LINC02583 | long intergenic non-protein coding RNA 2583 |
|  | chr2 | 151176 | ERFE | erythroferrone |
|  | chr3 | 401106 | LINC00884 | long intergenic non-protein coding RNA 884 |
|  | chr3 | 285224 | DNAJB8-AS1 | DNAJB8 antisense RNA 1 |
|  | chr3 | 1618 | DAZL | deleted in azoospermia like |
|  | chr4 | 285505 | FAM198B-AS1 | FAM198B antisense RNA 1 |
|  | chr4 | 27146 | FAM184B | family with sequence similarity 184 member B |
|  | chr4 | 644962 | TNRC18P1 | trinucleotide repeat containing 18 pseudogene 1 |
|  | chr5 | 373863 | DND1 | DND microRNA-mediated repression inhibitor 1 |
|  | chr5 | 54514 | DDX4 | DEAD-box helicase 4 |
|  | chr5 | 728637 | MEIKIN | meiotic kinetochore factor |
|  | chr5 | 79888 | LPCAT1 | lysophosphatidylcholine acyltransferase 1 |
|  | chr5 | 11336 | EXOC3 | exocyst complex component 3 |
|  | chr5 | 815 | CAMK2A | calcium/calmodulin dependent protein kinase II alpha |
|  | chr5 | 100302156 | MIR1229 | microRNA 1229 |
|  | chr5 | 2149 | F2R | coagulation factor II thrombin receptor |
|  | chr6 | 8871 | SYNJ2 | synaptojanin 2 |
|  | chr6 | 102724511 | LOC102724511 | uncharacterized LOC102724511 |
|  | chr6 | 6582 | SLC22A2 | solute carrier family 22 member 2 |
|  | chr6 | 7148 | TNXB | tenascin XB |
|  | chr6 | 6581 | SLC22A3 | solute carrier family 22 member 3 |
|  | chr6 | 4201 | MEA1 | male-enhanced antigen 1 |
|  | chr6 | 401289 | LINC01624 | long intergenic non-protein coding RNA 1624 |
|  | chr7 | 442497 | LOC442497 | uncharacterized LOC442497 |
|  | chr7 | 100616116 | MIR4648 | microRNA 4648 |
|  | chr7 | 109623488 | SNORA114 | small nucleolar RNA, H/ACA box 114 |
|  | chr7 | 100616160 | MIR4655 | microRNA 4655 |
|  | chr7 | 401357 | LOC401357 | uncharacterized LOC401357 |
|  | chr7 | 768213 | MIR671 | microRNA 671 |
|  | chr7 | 644794 | LINC02604 | long intergenic non-protein coding RNA 2604 |
|  | chr8 | 254896 | LOC254896 | uncharacterized LOC254896 |
|  | chr8 | 100288527 | REXO1L2P | REXO1 like 2, pseudogene |
|  | chr8 | 51001 | MTERF3 | mitochondrial transcription termination factor 3 |
|  | chr8 | 2515 | ADAM2 | ADAM metallopeptidase domain 2 |
|  | chr8 | 100288527 | REXO1L2P | REXO1 like 2, pseudogene |
|  | chr8 | 100288527 | REXO1L2P | REXO1 like 2, pseudogene |
|  | chr8 | 100288527 | REXO1L2P | REXO1 like 2, pseudogene |
|  | chr8 | 100288527 | REXO1L2P | REXO1 like 2, pseudogene |
|  | chr8 | 64236 | PDLIM2 | PDZ and LIM domain 2 |
|  | chr8 | 340393 | TMEM249 | transmembrane protein 249 |
|  | chr9 | 100616242 | MIR4673 | microRNA 4673 |
|  | chr9 | 5730 | PTGDS | prostaglandin D2 synthase |
|  | chr9 | 401561 | LINC01451 | long intergenic non-protein coding RNA 1451 |
|  | chr10 | 84287 | ZDHHC16 | zinc finger DHHC-type containing 16 |
|  | chr10 | 5214 | PFKP | phosphofructokinase, platelet |
|  | chr10 | 101929574 | LOC101929574 | uncharacterized LOC101929574 |
|  | chr10 | 22876 | INPP5F | inositol polyphosphate-5-phosphatase F |
|  | chr11 | 1410 | CRYAB | crystallin alpha B |
|  | chr11 | 100287837 | LOC100287837 | uncharacterized LOC100287837 |
|  | chr11 | 11187 | PKP3 | plakophilin 3 |
|  | chr11 | 105369343 | MIR194-2HG | MIR194-2 host gene |
|  | chr11 | 100616284 | MIR4489 | microRNA 4489 |
|  | chr12 | 574449 | MIR492 | microRNA 492 |
|  | chr12 | 105369595 | LOC105369595 | uncharacterized LOC105369595 |
|  | chr12 | 59341 | TRPV4 | transient receptor potential cation channel subfamily V member 4 |
|  | chr12 | 59341 | TRPV4 | transient receptor potential cation channel subfamily V member 4 |
|  | chr12 | 23710 | GABARAPL1 | GABA type A receptor associated protein like 1 |
|  | chr12 | 359 | AQP2 | aquaporin 2 |
|  | chr12 | 1967 | EIF2B1 | eukaryotic translation initiation factor 2B subunit alpha |
|  | chr13 | 729250 | PRR20E | proline rich 20E |
|  | chr13 | 102723345 | LOC102723345 | uncharacterized LOC102723345 |
|  | chr14 | 122616 | CLBA1 | clathrin binding box of aftiphilin containing 1 |
|  | chr14 | 84312 | BRMS1L | BRMS1 like transcriptional repressor |
|  | chr14 | 101929634 | LINC02280 | long intergenic non-protein coding RNA 2280 |
|  | chr14 | 93487 | MAPK1IP1L | mitogen-activated protein kinase 1 interacting protein 1 like |
|  | chr16 | 606724 | LOC606724 | coronin 1A pseudogene |
|  | chr16 | 100423012 | MIR3177 | microRNA 3177 |
|  | chr16 | 101059953 | NPIPA8 | nuclear pore complex interacting protein family member A8 |
|  | chr16 | 348174 | CLEC18A | C-type lectin domain family 18 member A |
|  | chr16 | 89941 | RHOT2 | ras homolog family member T2 |
|  | chr16 | 101927793 | LOC101927793 | uncharacterized LOC101927793 |
|  | chr16 | 104644205 | ENPP7P13 | ectonucleotide pyrophosphatase/phosphodiesterase 7 pseudogene 13 |
|  | chr17 | 100996842 | LOC100996842 | uncharacterized LOC100996842 |
|  | chr17 | 84440 | RAB11FIP4 | RAB11 family interacting protein 4 |
|  | chr17 | 284123 | FAM27E5 | family with sequence similarity E5 |
|  | chr17 | 100616170 | MIR4739 | microRNA 4739 |
|  | chr17 | 29 | ABR | ABR activator of RhoGEF and GTPase |
|  | chr17 | 6329 | SCN4A | sodium voltage-gated channel alpha subunit 4 |
|  | chr17 | 5986 | RFNG | RFNG O-fucosylpeptide 3-beta-N-acetylglucosaminyltransferase |
|  | chr17 | 102466911 | MIR6785 | microRNA 6785 |
|  | chr17 | 146849 | CCDC42 | coiled-coil domain containing 42 |
|  | chr17 | 643008 | SMIM5 | small integral membrane protein 5 |
|  | chr18 | 105372179 | LOC105372179 | uncharacterized LOC105372179 |
|  | chr18 | 102724130 | LINC02564 | long intergenic non-protein coding RNA 2564 |
|  | chr18 | 64693 | CTAGE1 | cutaneous T cell lymphoma-associated antigen 1 |
|  | chr18 | 4152 | MBD1 | methyl-CpG binding domain protein 1 |
|  | chr19 | 55422 | ZNF331 | zinc finger protein 331 |
|  | chr19 | 57479 | PRR12 | proline rich 12 |
|  | chr19 | 374887 | YJEFN3 | YjeF N-terminal domain containing 3 |
|  | chr19 | 102465856 | MIR7974 | microRNA 7974 |
|  | chr19 | 102465480 | MIR6800 | microRNA 6800 |
|  | chr19 | 100616314 | MIR4750 | microRNA 4750 |
|  | chr19 | 51298 | THEG | theg spermatid protein |
|  | chr20 | 100422945 | MIR4326 | microRNA 4326 |
|  | chr20 | 101928604 | ZBTB46-AS1 | ZBTB46 antisense RNA 1 |
|  | chr20 | 128876 | FAM83C | family with sequence similarity 83 member C |
|  | chr20 | 55861 | DBNDD2 | dysbindin domain containing 2 |
|  | chr20 | 100506384 | LOC100506384 | uncharacterized LOC100506384 |
|  | chr20 | 406923 | MIR133A2 | microRNA 133a-2 |
|  | chr20 | 728882 | FAM182B | family with sequence similarity 182 member B |
|  | chr21 | 103504727 | MIR6724-2 | microRNA 6724-2 |
|  | chr22 | 613 | BCR | BCR activator of RhoGEF and GTPase |
|  | chr22 | 29797 | POM121L8P | POM121 transmembrane nucleoporin like 8, pseudogene |
|  | chr22 | 646074 | POM121L10P | POM121 transmembrane nucleoporin like 10, pseudogene |
|  | chr22 | 100616414 | MIR4761 | microRNA 4761 |
|  | chr22 | 400931 | MIRLET7BHG | MIRLET7B host gene |
|  | chr22 | 25812 | POM121L1P | POM121 transmembrane nucleoporin like 1, pseudogene |
|  | chr22 | 55586 | MIOX | myo-inositol oxygenase |
|  | chrX | 266740 | MAGEA2B | MAGE family member A2B |
|  | chrX | 100130302 | SUPT20HL1 | SUPT20H like 1 |
|  | chrX | 389874 | ZCCHC13 | zinc finger CCHC-type containing 13 |
|  | chrX | 5634 | PRPS2 | phosphoribosyl pyrophosphate synthetase 2 |
|  | chrX | 3476 | IGBP1 | immunoglobulin binding protein 1 |
|  | chrX | 4111 | MAGEA12 | MAGE family member A12 |
|  | chrX | 474381 | H2AFB2 | H2A histone family member B2 |
|  | chrX | 100132994 | CXorf49B | chromosome X open reading frame 49B |
|  | chrX | 4101 | MAGEA2 | MAGE family member A2 |
